# Stimulus‐Responsive Hydrogels as Drug Delivery Systems for Inflammation Targeted Therapy

**DOI:** 10.1002/advs.202306152

**Published:** 2023-11-20

**Authors:** Haoyu Yu, Rongyao Gao, Yuxin Liu, Limin Fu, Jing Zhou, Luoyuan Li

**Affiliations:** ^1^ The Eighth Affiliated Hospital Sun Yat‐sen University Shenzhen Guangdong 518033 P. R. China; ^2^ Department of Chemistry Renmin University of China Beijing 100872 P. R. China; ^3^ Department of Biomolecular Systems Max‐Planck Institute of Colloids and Interfaces 14476 Potsdam Germany; ^4^ Department of Chemistry Capital Normal University Beijing 100048 P. R. China

**Keywords:** drug delivery, immune responses, inflammation targeted therapy, inflammatory microenvironments, stimulus‐responsive hydrogels

## Abstract

Deregulated inflammations induced by various factors are one of the most common diseases in people's daily life, while severe inflammation can even lead to death. Thus, the efficient treatment of inflammation has always been the hot topic in the research of medicine. In the past decades, as a potential biomaterial, stimuli‐responsive hydrogels have been a focus of attention for the inflammation treatment due to their excellent biocompatibility and design flexibility. Recently, thanks to the rapid development of nanotechnology and material science, more and more efforts have been made to develop safer, more personal and more effective hydrogels for the therapy of some frequent but tough inflammations such as sepsis, rheumatoid arthritis, osteoarthritis, periodontitis, and ulcerative colitis. Herein, from recent studies and articles, the conventional and emerging hydrogels in the delivery of anti‐inflammatory drugs and the therapy for various inflammations are summarized. And their prospects of clinical translation and future development are also discussed in further detail.

## Graphical abstract

Deregulated Inflammation is one of the most common diseases in human health, and its management remains a significant challenge. Notably, stimulus‐responsive hydrogels have great potential to break through the predicament faced by severe inflammations treatment. This review highlights on the recent advances of stimulus‐responsive hydrogels for inflammation therapy and challenges in this area, to provide incentive for their further development.

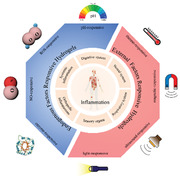

## 1 Introduction

Inflammation is an internal defensive response induced by immune cells and cytokines, which aimed at repairing injured tissues and clearing various damage factors such as pathogens. Any factor leads to cell or tissue damage could cause inflammations.^[^ ^1^ ^]^ Normally, the procedures of inflammation are as follows.^[^ ^2^ ^]^ i) Rapid introduction phase. First, the danger signals are released from damaged cells or pathogens. The around immune cells, such as macrophage and circulating neutrophils, are activated and then release inflammatory mediators to recruit more immune cells to the inflamed site. Then, transmigration of leukocyte is triggered, which is an essential characteristic of inflammatory response. In the first 24 h, neutrophils aggregate in the inflamed site, release tumor necrosis factor (TNF) to increase the permeability of vascular. Then, in the next 24 to 48 h, most of the neutrophils have been apoptosis, while monocytes are attracted to the inflammatory site by the chemokines produced by neutrophils. Monocytes will release proinflammatory cytokines, such as reactive oxygen species (ROS), to promote inflammatory response in a further degree. Meanwhile, immune cells will clear dead cells and inflammatory agents by phagocytosis or immune responses. ii) Resolution phase. In this stage, the anti‐inflammatory cytokines are released by immune cells, like interlukin‐10 (IL‐10) which released by M2 phenotype macrophages, to induce the regeneration of damaged tissues. Then the inflammation response will alleviate and finally get recovery.

**Figure 1:**
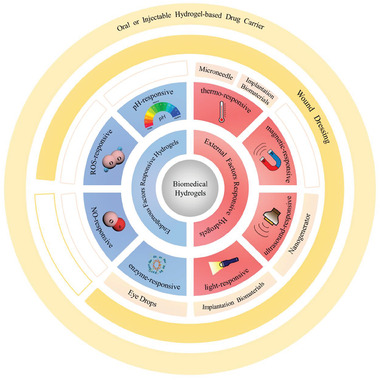
Classification of stimulus‐responsive hydrogels for various inflammation therapy.

As the above, inflammatory response is beneficial to people's health because of its promotion for the recovery of damage or clearance of pathogens. However, for some reasons, like the innate immunity deficiency, the resolution phase could be uncontrolled and may lead to local or systemic dysregulated inflammations, which accompanied with redness, swelling, heat, pain, and dysfunction ordinarily.^[^ ^3^ ^]^ The administration of anti‐inflammatory drugs is the most often used therapy method in the clinical treatment. But there are also many limitations of these drugs, particularly the poor solubility, less permeability, and short retention time, and some of them even have unfavorable side effects.^[^ ^4^ ^]^ To address these limitations, many efforts have been dedicated to the design and investigate of drug carriers, wound dressing, and composite drug release systems such as micro/nanoparticles, exosomes, and biomimetic materials.^[^ ^5^ ^]^ Among these researches, smart hydrogel which possesses good biocompatibility, design flexibility, and unique physical and chemical properties has been a kind of potential and effective biomaterial to treat inflammations. According to their action mechanism, these hydrogels could be mainly divided into endogenous factor (e.g., pH, ROS, biomolecule) responsive hydrogel, external factor (e.g., light, ultrasound) responsive hydrogel, and multifactor‐responsive hydrogel (**Figure** **1**). In this review, we first introduced some typical and common inflammations as well as their symptoms, pathogenesis, and therapy. And next, commonly used materials for the preparation of smart hydrogels are showcased and their representative researches are also mentioned simultaneously. Then we concluded the latest development and applications of the above three categories of stimuli‐responsive hydrogels. Finally, the envisions and challenges in this filed were also discussed.

## 2 Various Types of Inflammations Treated with Stimulus‐Responsive Hydrogels

According to persistent time and pathological process, dysregulated inflammation usually could be divided into acute inflammation and chronic inflammation (**Table** **1**).^[^ ^2^ ^a]^ Acute inflammation is a rapid response of the immune system to inflammatory factors, which aims at transporting immune cells or plasma proteins to the inflamed sites and eliminating these inflammatory factors eventually.^[^ ^2^ ^c]^ This process is generally accompanied with vascular and leukocyte response and the persistent time often ranges from a few days to a month,^[^ ^6^ ^]^ such as acute appendicitis, acute gastroenteritis, and acute pneumonia. Most acute inflammations could get recovered by effective therapy but some of them may be prolonged to a chronic inflammation under these conditions:^[^ ^2^ ^c^ ^7^ ^]^ i) the pathogens are difficult to be eliminated for some reasons. For example, *Escherichia coli* may lead to chronic enteritis. ii) Prolonged exposure to toxic factors. For example, long‐term exposure to silicon dioxide (SiO_2_) will increase the probability of silicosis. iii) Autoimmune disease induced by the out of control of the immune system is represented by systemic lupus erythematosus (SLE). Chronic inflammations could dure for months or even several decades, which are extremely harmful to people's health.

**Table 1:** Classification of inflammations.

Type	Characteristics	Clinical treatments		Common types (most common pathogenic factors)
Acute inflammations	Short‐duration. Usually persists for a few days. No more than a month. Infiltrating cells are mainly granulocyte, based on different inflammatory factors, including neutrophil (infection of bacteria), basophils and eosinophils (allergy). Mainly exudative inflammations.	Acute inflammation with mild symptoms can be recovered by the regulation of immune system. Administration of anti‐inflammatory drugs. (oral delivery or injection) Surgery. Other treatments for specific acute inflammations.		Acute gastroenteritis (salmonella) COVID‐19 (corona virus‐19) Acute pharyngitis (allergy) Acute appendicitis (luminal obstruction) Acute myocarditis (coxsackievirus B) Acute viral hepatitis type B (hepatitis B) Poliomyelitis (poliovirus)
Chronic inflammations	Long‐duration. Accompanied with intermittent recurrent acute inflammations. Ranging from months to years, even life‐long. Infiltrating cells are mainly monocytes, plasma cells, and lymphocytes. Mainly proliferative inflammations. No obvious symptoms during the interphase of inflammation.	Attack stage Remission stage	Treating the same as acute inflammations. Continued drug treatments to avoid relapse. Improvement of lifestyle could be helpful for some specific chronic inflammations.	Chronic gastritis (gastric mucosal damage) Chronic bronchitis (noxious gas, virus, and bacteria) Ulcerative colitis (still unclear) Periodontitis (dental plaque) Rheumatoid arthritis (still uncertain) Chronic nephritis (glomerulopathy) Bronchial asthma (heredity) Allergic rhinitis (interaction between gene and environment)
Infectious inflammations	Induced by biotic factors, such as bacteria, virus, parasite, spirochete, fungus, mycoplasma, Chlamydia, and Rickettsia.	Injection or oral delivery of drugs for specific pathogens. Surgery may be needed in severe cases.		Septicemia (infection of various bacteria) Spotted fever (rickettsia) Mycoplasma pneumoniae (mycoplasma) Malaria (plasmodium) Sepsis (infection of microorganism)
Aseptic inflammations	Induced by abiotic factors, such as abnormal temperature, ultraviolet, chemical/biotic agents, mechanical trauma, radioactive rays, undesirable immune response, and tissue necrosis.	Injection or oral delivery of anti‐inflammatory drugs. Surgery may be needed in severe cases. Other treatments for specific aseptic inflammations.		Chronic gastritis (gastric mucosal damage) Plantar fasciitis (overuse injuries) Gout (hyperuricemia) Osteoarthritis (aging and obesity) Systemic lupus erythematosus (autoimmunity)

Besides, as shown in Table 1, based on the pathogenic factors, inflammations could be divided into infectious inflammations and aseptic inflammations (noninfectious inflammations). Infectious inflammations are induced by biological factors like bacteria, virus, and mycoplasma. On the contrary, aseptic inflammations are triggered by physical, chemical or other noninfectious factors, like ultraviolate, toxic gas, sprain, and dysimmunity, and have similar symptoms to infectious inflammations.^[^ ^8^ ^]^ Therefore, clinical treatment of infectious and aseptic inflammations is totally different. For instance, antibiotics could be used for the dampening of bacteria‐induced inflammation but useless for tenosynovitis.

Inflammatory microenvironments (IMEs) are formed by immune cells, inflammation‐related enzymes, and inflammatory mediators.^[^ ^5^ ^]^ Hence, these factors or biomarkers and their unique features provide opportunities for the precise delivery of drugs or local therapy, also various trigger factor for stimuli‐responsive hydrogels (**Table** **2**). For instance, inflammations lead tissues in inflammatory site to increased metabolic activity, which may consequently cause hypoxia. Then, abundant lactic acids are produced around these cells, thereby making the IMEs acidic.^[^ ^9^ ^]^ In light of this, a great deal of pH‐responsive drug delivery platform has been developed, such as various nanoparticles and hydrogels.^[^ ^10^ ^]^

**Table 2:** Common components in IMEs.

	Representative substances		Produced by	Normal functions		Features or effects during dysregulated inflammations	Refs.
Proinflammatory enzymes	Alkaline phosphatases (ALPs)		Macrophages neutrophils, etc.	Hydrolyzing phosphoester bonds.		Overexpressed in inflammatory sites. Protecting tissues.	[11]
	Human neutrophil elastase (HNE)		Neutrophils	Hydrolyzing some proteins such as collagen, elastin, and fibronectin. Playing a crucial role in the secretion of inflammatory mediators which will recruit more immune cells to IMEs.		Overexpressed in inflammatory sites. Degrading connective cells thus leading to tissue damage. Contributing to persistent inflammations.	[12]
	Matrix metalloproteinases (MMPs)		Macrophages, lymphocytes, endothelial cells, neutrophils, etc.	Hydrolyzing proteins like collagens and glycoproteins in extracellular matrix. Promoting cell proliferation, migration, and differentiation. Modulating cell signal.		Overexpressed in inflammatory sites. Contributing to disruption and regeneration of inflammatory tissues.	[13]
Inflammatory cells	Macrophages		Differentiated from monocytes	M1	Inducing inflammation by releasing inflammatory mediators, such as IL‐6, IL‐1β, TNF‐α. Clearing proinflammatory factors by necrosis. Presenting antigen to T cells.	The balance of M1/M2 is disrupted by excessive M1, this could lead to chronic inflammation.	[14]
				M2	Removing debris and apoptotic cells. Suppressing inflammation by releasing anti‐inflammatory cytokines such as TGF‐β, IL‐10.		
Inflammatory mediators	Reactive oxygen species (ROS, represented by H_2_O_2_)		Cellular metabolism	A small amount in normal tissues, usually acting as cell signals molecules. Released by macrophages to destroy pathogens.		High level ROS will attack cells indiscriminately, and are associated with chronic inflammation. Leading to oxidative stress.	[15]
	Reactive nitrogen species (RNS, represented by NO)		Cellular metabolism	Destroying pathogens directly. Acting as cellular signaling messengers. Regulating the expression of some proteins.		High level RNS will attack DNA, RNA, and proteins indiscriminately, which is deleterious for cells. Leading to oxidative stress.	[15, 16]
	Tumor necrosis factors (TNF‐α, TNF‐β)		Macrophages (TNF‐α) Activated T cells, etc. (TNF‐β)	Acting as proinflammatory factor. Activating endothelial cells which will enhance the permeability of microvascular. Causing the necrosis of tumor cells. Promoting the secretion of other proinflammatory factors like IL‐1, IL‐6, IL‐12.		Overexpressed in inflammatory sites. TNF is a key inflammatory factor, which will stimulate the secretion of other inflammatory cytokines and chemokines.	[17]
	Interleukin (IL)	IL‐1	Macrophages	Existing as IL‐1α and IL‐1β. Involving in immune regulations.		Overexpressed in inflammatory sites. Leading to fever.	[18]
		IL‐6	Macrophages, Th2 cells	A key proinflammatory factor which stimulates various immune cells and inducing acute inflammation.		Overexpressed in inflammatory sites. Long‐term high‐level IL‐6 may lead to sepsis. Usually regarded as an indicator of systemic Inflammations in the clinic.	
		IL‐10	Monocytes Th2 cells Mast cells	Anti‐inflammatory factor, suppressing the immune response of monocytes.		(Overexpressed) Leading to immune deficiency, resulting in the tumor growth or bacterial infection. (Less expressed) Leading to chronic inflammations.	

**Figure 2:**
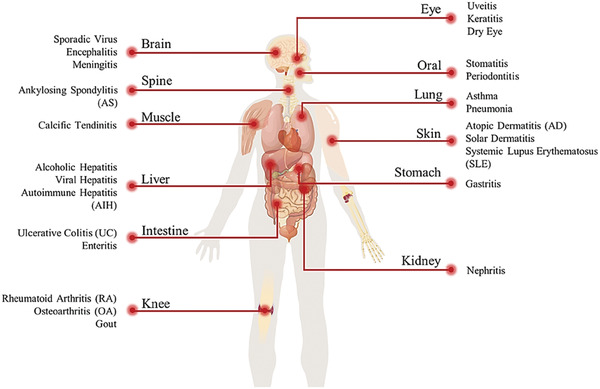
Common or typical inflammations in various organs or tissues.

In this section, according to the main organ and body parts involved, we would introduce several kinds of inflammatory diseases, including their common symptoms, pathology, strategies, and issues during their current therapy, briefly (**Figure** **2**). And recent design of hydrogels which is used for the management or treatment of these inflammations is also mentioned.

### 2.1 Orthopedic inflammation Treated with Cytokine‐Responsive Hydrogels

Most orthopedic inflammation is chronic and caused by a variety of complex factors, and in which cytokines play crucial roles. Therefore, the construction of cytokine‐responsive hydrogels has a broad prospect for the treatment of orthopedic diseases.

#### 2.1.1 Rheumatoid Arthritis (RA)

RA is a chronic and systematic autoimmune disease, which is characterized by synovitis, morning stiffness, and usually accompanied with serious inflammatory response, bone or cartilage damage, and symmetry joint deformation and swelling.^[^ ^19^ ^]^ Besides, RA could also trigger many complications such as keratitis, Caplan syndrome, pneumoconiosis, endocarditis, while most of the RA dead cases are caused by cardiovascular or lung complications.^[^ ^20^ ^]^ Medical research has verified that the occurrence of RA is closely related to many factors:^[^ ^21^ ^]^ i) the induction of bacteria or virus infection, ii) sex hormone, which also makes the female more susceptible to RA, iii) genetic factors, which accounts for ≈60% of the patients, and iv) inhabits and living environment like that smoking could increase the risk of RA.

**Figure 3:**
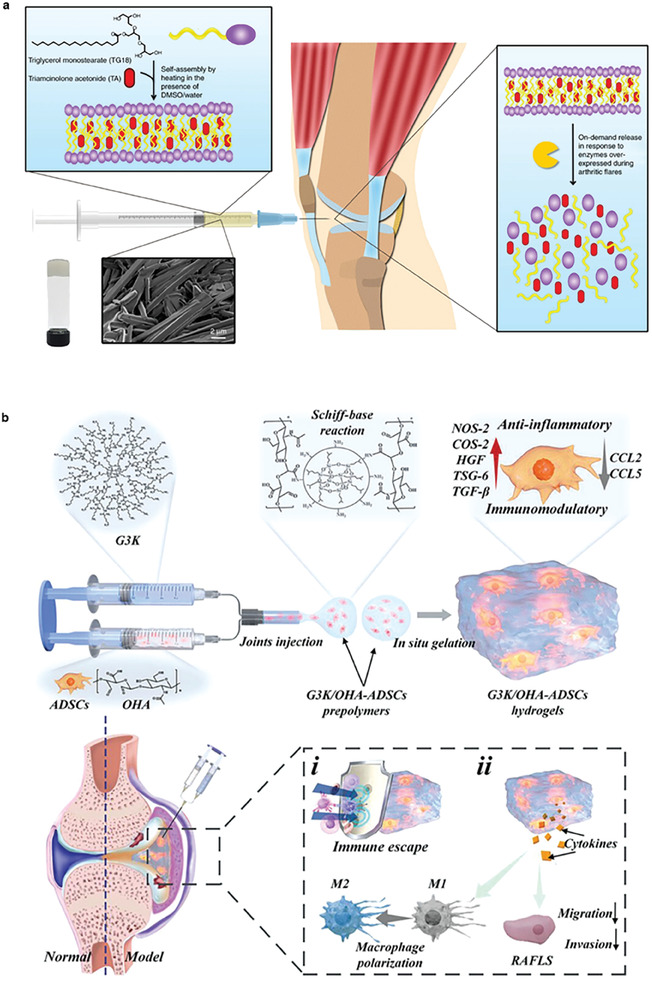
a) An injectable self‐assembly hydrogel that responsive to MMPs in inflammatory joint. Reproduced with permission.^[^ ^25^ ^]^ Copyright 2018, Springer Nature. b) A novel extracelluar matrix‐inspired injectable hydrogel for adipose‐derived stem cells encapsulation and delivery to treat RA. Reproduced with permission.^[^ ^25^ ^]^ Copyright 2023, John Wiley & Sons.

Traditional clinical treatment methods of RA could be divided into surgery and drug administration. Surgery strategy could improve the quality of life (QOL) of patients by having joint replacement or synovectomy, but not helpful for the recovery and control of RA progression. Drug administration is the most common clinical therapeutic strategy in the treatment of RA. And the drugs used to manage RA could be classified into three categories.^[^ ^22^ ^]^ i) Nonsteroidal anti‐inflammatory drugs (NSAIDs), which are represented by ibuprofen (IBF), diclofenac sodium (DS), and celecoxib. NSAIDs focus on the alleviation of RA syndromes but are almost useless for the control of RA progression. Thus, patients need ii) disease‐modifying antirheumatic drugs (DMARDs), which could also be further classified into conventional synthetic DMARDs (csDMARDs), targeted synthetic DMARDs, and biological DMARDs. While csDMARDs, including methotrexate (MTX), hydroxychloroquine (HCQ), and sulfasalazine (SSZ), are the first line medicines in clinical therapy. Although DMARDs could suppress the deterioration of RA, their different serious and undesired side effects greatly limit their clinical long‐term administration and they usually take a longer time to get effective after administration. iii) Glucocorticoid drugs (GCs), represented by dexamethasone (DEX), which possess both excellent anti‐inflammatory effect and RA suppression capacity. However, GCs are toxic and addictive, so its application should follow the criteria of modicum and short‐term. According to the recommendations of RA management which was published by European League Against Rheumatism in 2019, the combination use of DMARDs, NSAIDs, and GCs make up the key of RA control (**Figure** **3**).^[^ ^23^ ^]^ Ordinarily, the clinical drug treatment of RA is based on the combination of various drugs. The NSAIDs and GCs are used for the alleviation of acute syndromes, and DMARDs are applied for long‐term management.

And besides these conventional therapeutic strategies, there are also many novel methods to treat RA including stem cell therapy, and immune therapy, which has been used in clinical practice.^[^ ^24^ ^]^ To minimize the undesired side effects and improve therapeutic effects, injectable hydrogels, which are responsive to various biomarkers in RA‐induced joints were presented to carry drug molecules or stem cells for RA management.^[^ ^25^ ^]^ But the traditional therapeutic strategies are still the mainstream of RA management as well as the conventional drug therapy also face challenges due to the inherent limitations of the first line drugs. For example, the insolubility of MTX in water hinders its therapeutic effect and the serious side effect of unselective NSAIDs and GCs limit their long‐term administration. Moreover, the ambiguity of the pathogenic mechanism of RA makes this disease incurable. And the current treatment still focuses on relieving symptoms and delaying the progression of RA. Hence, the research about RA pathology also needs to be broken through urgently.

#### 2.1.2 Osteoarthritis (OA)

OA is also a common arthritis, which is accompanied with sharp ache, shorter morning stiffness that compared with RA patients and joint degeneration. Aging, joint injury, mechanical stress, genetic factors, sex, and even obesity were thought to be the culprits for the occurrence of OA.^[^ ^26^ ^]^ Chondrocyte in joint is responsible for cartilage‐related physiological activity. These specific cells exist in a strong gelatinous extracellular matrix, which contains a lot of proteoglycans like hyaluronic acid (HA) and chondroitin sulfate (CS) and maintain the toughness and flexibility of joint. But during the inflammatory response in joint, the production of IL‐1, IL‐6, and TNF by immune cells would influence the activity of related enzymes and then destroy the balance between the catabolic and anabolic activity of chondrocyte, finally causing the cartilage loss.^[^ ^27^ ^]^

Nowadays, because of the irreversibility of chondrocyte reduction and cartilage loss, clinical treatment for OA mainly aims at the symptom remission and QOL improving. For patients with mild symptoms, nonmedical approaches like losing weight and moderate exercise could be an appropriate choice. As for medical means, similar to the management of RA, the therapeutic strategy of OA could also be divided into two categories: i) pharmacological approaches, such as the administration of NSAIDs and injection of GCs or HA;^[^ ^28^ ^]^ ii) surgery approaches, represented by joint replacement. Besides, researchers also proposed the intra‐articular injection of stem cells to promote cartilage repair.^[^ ^29^ ^]^ Similar to the hydrogels that used for RA treatment, various injectable hydrogels with stimuli responsibility, like thermal sensitivity and ROS sensitivity, were proposed as drug carriers or tissue scaffolds to realize OA relief or promote cartilage regeneration. Especially, as the crucial component of synovial fluid, HA‐based hydrogels received a lot of attention from researchers.^[^ ^30^ ^]^

#### 2.1.3 Gout

Gout is a common arthritis caused by hyperuricemia, while the occurrence of hyperuricemia could be further attributed to the increased production of purine, the precursor of uric acid, or decreased metabolism of uric acid itself. The needle‐like monosodium urate crystals, which deposit in joints, especially the first metatarsal joint of foot, recruit a number of immune cells so that induce serious inflammatory response.^[^ ^31^ ^]^ Gouty arthritis generally comes with swelling, red as well as sharp ache during acute flare‐ups, thus putting harmful effect on patients’ lives. Tophus is usually found in severe cases.

**Figure 4:**
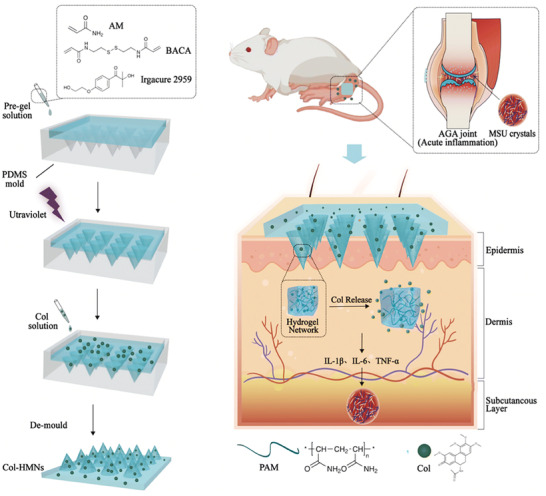
A novel hydrogel microneedle patch for gout management. Reproduced with permission.^[^ ^32^ ^]^ Copyright 2023, Royal Society of Chemistry.

The treatment of acute gouty arthritis mainly focuses on the fast relief of pain and swelling. Colchicine, NSAIDs like DS and GCs are frequently‐used to help with gout remission. As to the management of chronic gouty arthritis, diet modification and exercise are usually the first choice. Inhibitors like allopurinol, which could prevent the transformation of purines to uric acid by inhibiting the activity of xanthine oxidase, are also applied to control the uric acid level in blood. Moreover, researchers investigated a colchicine‐loaded hydrogel microneedle for acute gouty arthritis management and were verified effective in mouse models (**Figure** **4**).^[^ ^32^ ^]^

#### 2.1.4 Ankylosing Spondylitis (AS)

AS is a rheumatic disease and characterized by rigid spine. The same as other autoimmune disease, the mechanism of AS is not super clear but closely related to the immune cells induced attack on the collagens I and II, which were replaced by fibrin eventually in vertebral joints.^[^ ^33^ ^]^ AS not only cause spine damage but also trigger inflammations in other organs such as uveitis and enthesitis, as well as fever, stiffness, and sharp ache in backbone, knee, and hip. Drugs used for RA treatment including NSAIDs (e.g., indomethacin) and csDMARDs (e.g., MTX and SSZ) are effective for the management of AS. Besides, moderate exercise and physical method are also beneficial for the relief of AS.^[^ ^33^ ^]^ The damaged hip and knee joints could be repaired by surgery but not suitable for spine because of high risk. Recently, various drug‐loaded hydrogel patches were developed and applied for the management of AS.^[^ ^34^ ^]^

### 2.2 Inflammations in Digestive System Treated with pH‐Responsive Hydrogels

Inflammations in digestive system are often accompanied by the pH changes in different bodily fluids, which generally make it difficult for the drugs to achieve better therapeutic effects. Reasonable selections for pH‐responsive hydrogels are helpful to improve drug availability and treatment status.

#### 2.2.1 Ulcer Colitis (UC)

The common symptoms of UC include serious pain and diarrhea that sometimes with blood in stool.^[^ ^35^ ^]^ Although the precise pathological mechanism of UC is still unknown, it is generally thought that the UC is constitutionally an autoimmune chronic disease with genetic predisposition and was also induced by the combination of various environmental stimuli like diet, stress, and intestinal flora. And cytotoxic T cells were thought responsible for the form of ulcers along the colon or large intestine by inducing the mucosal destruction and attack on epithelium cells.^[^ ^36^ ^]^

Based on the severity, current treatment toward UC could be toughly summarized into three steps. i) Drug administration, which aims at the suppression of inflammation. SSZ and mesalamine are frequently used type in clinical treatment. ii) Immunosuppressant drugs such as GCs and azathioprine. iii) Surgery. Colotomy could be performed when the state further deteriorated. Besides, researchers have developed a series of hydrogel drug carriers, which using intestinal temperature, pH, or enzymes as switches to achieve precise drug delivery by oral administration.^[^ ^37^ ^]^

#### 2.2.2 Autoimmune Hepatitis (AIH)

AIH is an autoimmune inflammation that young women are more susceptible to. The clear pathogenesis of AIH is also uncertain but generally was thought related to the combination of genetic factors and environmental triggers and predominantly affects women.^[^ ^38^ ^]^ The symptoms of AIH are also not exactly the same based on individuals. Patients could be completely asymptomatic and could also present liver cirrhosis or acute hepatic damage.^[^ ^39^ ^]^ As to treatment, the pharmaceutical methods used for AIH therapy are similar to that of UC. Immunosuppressants like GCs and azathioprine are also effective and were regarded as first‐line drugs in clinical practice.^[^ ^40^ ^]^ Besides, liver transplantation was also considered when the above method is hardly helpful.

#### 2.2.3 Gastritis

Gastritis refers to the inflammation of gastric mucosa, which characterized by the destruction of mucosa and burning pain in stomach. It could be divided into acute gastritis and chronic gastritis according to the disease progression. Acute gastritis often caused by specific and clear factors, including infections, mechanical injury, and some medications. Acute gastritis is usually curable as timely and effective treatment was implemented.^[^ ^41^ ^]^

While the pathogenesis of chronic gastritis is usually more complex and not super clear. It has been demonstrated that the occurrence of chronic gastritis is related to various factors but mainly two categories: i) genetic factors, like autoimmune response; ii) environmental factors, such as diet, infection of *Helicobacter pylori* (Hp), and smoking. Chronic gastritis was also thought could be precancerous lesions.^[^ ^42^ ^]^ Since the complexity of the pathogenesis of chronic gastritis, the treatment is also different. For Hp‐induced gastritis, drug quadruple therapy should be first choice to remove bacteria. As to autoimmune gastritis, the supplementation of iron and vitamin B12 is necessary, while antacids and H2 receptor blockers is also need to protect the gastric mucosa from further destruction.^[^ ^43^ ^]^ Furthermore, patients with chronic gastritis should improve their lifestyle and have regular follow‐up.

### 2.3 Inflammations in Nervous System Treated with Chitosan‐Based Hydrogels

Inflammations in nervous system, or neuroinflammations, refers to the uncontrolled inflammatory response in brain or spinal cord, usually induced by the infection of bacteria or virus, mechanism injury, and neuropsychiatric disorders, could result in various neurodegenerative diseases (e.g., Alzheimer's disease and Parkinson's disease) and hinder the recovery of brain injury.^[^ ^44^ ^]^ Neuroinflammations always put great negative influence on the daily life of patients and their families. But there is no completely curable approach on the treatment of neuroinflammations and also no specific medicine. Current medical means mainly focus on delaying disease progression and improve symptoms, both of which are aiming at minimizing the adverse impact in patients’ life.

Therefore, effective suppression of neuroinflammations is crucial for the treatment of some tricky disease. Previous studies have shown that chitosan oligosaccharides play a protective role in ischemic brain injury in vitro, while the chitosan‐based hydrogels might provide some special advantages to treat inflammations in nervous system. Microglia‐mediated chronic neuroinflammation is thought play a critical role in the onset of Alzheimer's disease. Inspired by this, Dou and co‐workers proposed a chitosan‐based oxytocin‐loaded nanogel to prevent its occurrence by inhibiting the inflammatory response.^[^ ^45^ ^]^ Besides, some researchers were inspired by herbal medicine and combined them with novel biomaterials. For example, Wang and co‐workers attempted to load Rhein (4,5‐dihydroxyanthraquinone‐2‐carboxylic acid) into chitosan hydrogel or prepare Rhein hydrogel directly through self‐assembly, respectively, to improve its bioavailability and realize sustained release to control neuroinflammation effectively.^[^ ^46^ ^]^ Chitosan oligosaccharides (COS) play a protective role in ischemic brain injury in vitro.

### 2.4 Inflammations in Respiratory System Treated with Thermo/Enzyme‐Responsive Hydrogels

Respiratory inflammation often causes certain changes in body temperature and enzymes, leading to certain stress reactions. The thermo/enzyme‐responsive hydrogels as persistent drug delivery carriers are expected to propose more effective treatment schemes for the inflammations in respiratory system.

#### 2.4.1 Asthma

Asthma is a chronic inflammation in airways, which is caused by immune cells‐induced allergenic response. The pathogenesis of asthma is not clear but has familial transmissibility, and triggered by various environmental allergens, such as dust, smoke, and medications, which are different from person to person. The production of IL‐4 and IL‐5 from Th2 cells lead to many typical symptoms of asthma during acute flare‐ups, including mucus plugs, cough, and dyspnea.^[^ ^47^ ^]^ Without effective and timely management and treatment, asthma could further lead to lung edema or fibrosis.

As to the management of asthma, primarily, patients are supposed to avoid contacting with allergens in their daily life. Sever cases could avoid attack through drug administration like taking leukotriene, antagonists, and GCs. During acute flare‐ups, symptoms must be controlled in time by administrating some fast‐acting inhalants or drugs, such as bronchodilators and anticholinergics, or intravenous injection of GCs or magnesium sulfate. Oxygen therapy should be taken when further deterioration.^[^ ^48^ ^]^

#### 2.4.2 Pneumonia

**Figure 5:**
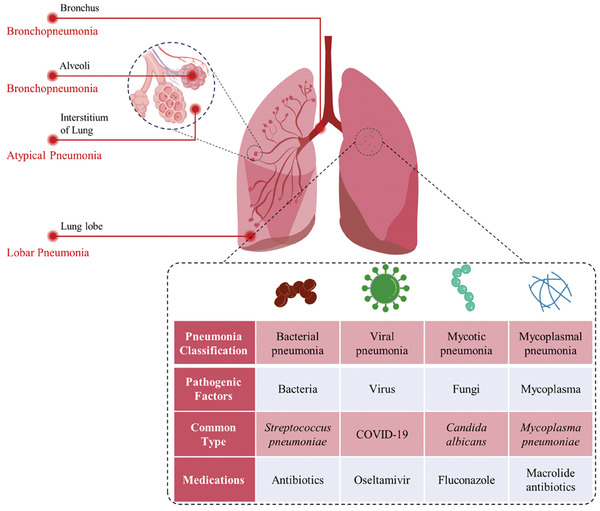
Classification of typical pneumonia based on pathogenic factors and lesion part.

Pneumonia is primarily caused by the infection of microorganisms in the lungs and results in the alveoli being filled with fluid.^[^ ^49^ ^]^ Since the complicated pathogenesis of pneumonia, this disease could be divided into several types according to different criteria (**Figure** **5**). According to the type of infectious microorganisms, pneumonia could be classified into i) bacterial pneumonia, ii) viral pneumonia, iii) mycotic pneumonia, and iv) mycoplasmal pneumonia. While bacterial pneumonia is the most common in adult.^[^ ^50^ ^]^ Based on the infected and lesion part, it could be divided into i) bronchopneumonia, which affects alveoli and bronchioles mainly; ii) lobar pneumonia, which infected the whole lobe of lung; iii) atypical pneumonia, whose infection mainly occurred outside the alveoli in the interstitium of lung. Patients with pneumonia often have cough, dyspnea, and sometimes systematic fever.

Antibiotics are the most frequently used type of medication for pneumonia treatment. Different suppressants are also prescribed based on various pathogenic factors. Additionally, cough suppressants and pain medications are applied to help with symptom relief.^[^ ^50^ ^]^

### 2.5 Inflammations in Sensory Organs Treated with pH/ROS‐Responsive Hydrogels

#### 2.5.1 Uveitis

Uveitis usually refers to the inflammatory lesions of iris, ciliary body, and choroid. Its treatment has been a hot topic in the medical field because of the variety and complex etiology. Although the precise pathogenesis is unclear, the factors that trigger uveitis can be divided into four categories: i) the infection of virus, bacteria, and rickettsia. For example, syphilitic uveitis was induced by the systemic infection of treponema pallidum; ii) mechanical injury. This type is represented by sympathetic ophthalmia;^[^ ^51^ ^]^ iii) autoimmune response caused by immune system; and iv) as a complication triggered by other inflammation such as ankylosing spondylitis.^[^ ^52^ ^]^ Besides the above, diet, stress, and environmental factors are also thought to be the part causation to uveitis.^[^ ^53^ ^]^ The symptom of uveitis sometimes exist huge difference due to the different variety, in which common symptoms including redness and pain of eye and diminution of vision.

Hence, the key to the therapy of uveitis is how to identify the cause accurately and suit the remedy to the case. The strategy of uveitis management could be classified into three categories. i) Medical treatment: eye drops were frequently used to control the inflammatory response. Though NSAIDs were effective while GCs such as the DEX and triamcinolone acetonide (TA) are the principal type of drugs for uveitis management.^[^ ^54^ ^]^ ii) Surgical treatment: removing nidus to prevent further deterioration. iii) Physical methods like hot compress are also effective for the relief of uveitis. And because of the abnormal amount of some specific enzymes in the eyes of uveitis patients, such as esterase and alkaline phosphatase (ALPs), the hydrogels activated by theses enzymes were investigated as eyedrops for uveitis therapy.^[^ ^55^ ^]^

#### 2.5.2 Atopic Dermatitis (AD)

AD, or eczema, is thought a genetic disease but not known too much about its pathogenesis. This disease is common among children and could last to adulthood. Typical symptoms include skin inflammation, pruritus, and xerosis, sometimes with lichenification in severe cases. Besides, erythematous papules could appear during acute flare‐ups. Although the pathogenesis of AD is thought mainly related to genetic factors, but other factors such as temperature and humidity, allergens, and mental stress could also induce AD.^[^ ^56^ ^]^

Both moderate and severe AD affect patients’ daily life greatly, even leads to social communication disorders and mental abnormalities. Therefore, the treatment of AD focuses on relieving symptoms and reducing the adverse influence on life.^[^ ^56^, ^57^ ^]^ i) Moisturizers play an important role in daily AD management. ii) During the acute flare‐ups of AD, topical corticosteroids or immunosuppressants are often used to suppress skin inflammation, oral antihistamines are administrated to control pruritus and topical antibiotics could be used when accompanied with bacterial infection. iii) Phototherapy is helpful for severe patients and those with recurrent attacks of AD. Currently, hydrogels used in the management and treatment of AD are mainly in the form of patches, which could prolong the action time through sustained drug release.^[^ ^58^ ^]^

#### 2.5.3 Periodontitis

Periodontitis is also a frequently researched inflammation with high morbidity, and could be further classified into aggressive periodontitis and chronic periodontitis based on the rate of progression. The main characteristics of periodontitis include inflammatory lesion of gingival soft tissue and alveolar bone resorption, which could leads to the formation of periodontal pocket.^[^ ^59^ ^]^ It is thought that the dental plaque contributes mostly to the onset of periodontitis.^[^ ^60^ ^]^ And smoking, dental calculus, injury, RA, leukemia, and diabetes could also be related to periodontitis.^[^ ^61^ ^]^

The therapy of periodontitis frequently follows the sequence of bacterial suppression, inflammation diminishing, and functional and morphological recovery of periodontal structure. The first and second aims could be accomplished simultaneously by removing dental calculus and using antibiotics like tetracycline and metronidazole. While the recovery of alveolar bone always be the crucial part of periodontitis treatment and also the most studied part. Presently, bone graft is commonly performed in clinical practice.^[^ ^60^ ^]^ Some type of biomaterials with the capacity to guide the regeneration of tissues is also applied. Besides, dental pulp stem cell also has great application potential in promoting alveolar bone regeneration because of the biosafety and low immunogenicity.^[^ ^62^ ^]^ After effective suppression of inflammatory response in oral, the damaged teeth could be repaired by filling or implantation to restore the function and morphology of teeth.

### 2.6 Systematic Inflammations Treated with Multistimulus‐Responsive Hydrogels

#### 2.6.1 Sepsis

Sepsis, characterized by serious systematic inflammatory response, is caused by the whole‐body infection of bacteria. Sepsis usually leads to low blood pressure, fever or hyperthermia and organ dysfunction and often triggers a lot of complications such as bacterial pneumonia and meningitis, resulting in shock and even death.^[^ ^63^ ^]^ There are three types of the most common pathogenic bacteria: i) gram positive bacteria (GPB) such as *Staphylococcus aureus*, ii) gram negative bacteria (GNB) such as *E. coli*, and iii) anaerobic bacteria like *Peptostreptococcus*. Immunocompromised people like newborns, elderly, and burn patients are more susceptible to sepsis.

The combination of various antibiotics should be used for initial patients. Then, the pathogenic bacteria are identified through blood culture and replaced the antibiotics with the most suitable type.^[^ ^64^ ^]^ For example, penicillin for GPB, gentamicin, and cephalosporin for GNB and metronidazole for anaerobic bacteria. In conclusion, the identification of pathogenic bacteria and selection of appropriate antibiotics are the keys to the treatment of sepsis.

#### 2.6.2 Systemic Lupus Erythematosus (SLE)

Similar to many diseases, SLE is an autoimmune disease and its precise mechanism and pathogenesis are still not understood. It was thought that the occurrence of SLE is closely related to the genetics and environment. i) First, the environmental stimuli, such as sunlight, smoke, and microorganisms, lead to the apoptosis of cells which release nuclear antigens. But these antigens and apoptotic bodies could not be cleared timely for some reasons. Then, ii) the immune cells produced a lot of antinuclear antibodies, which formed antigen–antibody complex through binding with nuclear antigens specially. Next, iii) the complex deposit to the vessel and circulate to skin, heart, joint, and eventually to the whole body. iv) The immune system recognizes the complex as foreign and triggered systematic inflammation. The above procedure was called type III hypersensitivity reaction, which may be the cause of most SLE cases.^[^ ^65^ ^]^ Besides environmental stimuli, sex hormones are also a trigger of SLE, which explains that women are more susceptible.

The symptoms of SLE are various and could be different according to individuals, but fever, rash, and arthritis are more common. In addition to no specific antibodies being found in SLE patients, the diagnosis of SLE gets particularly difficult and complicated.^[^ ^66^ ^]^

SLE is also characterized by the alternate of flare‐up periods and remittance period so patients should avoid to contact with the specific stimuli in their daily life to reduce the odds of onset. Since the symptoms are various and complicated, the pharmaceutical management of SLE is not always the same. Usually, GCs are used to relieve the inflammatory response and sometimes immunosuppressants like HCQ are also helpful.^[^ ^67^ ^]^

## 3 Commonly Used Materials in the Preparation of Stimulus‐Responsive Hydrogels

Generally, hydrogel systems are composed of hydrogel matrix and its contents (e.g., nanoparticles, drug molecules), which decide the characteristics of drug release system commonly. Hence, the applications of different hydrogel matrix usually depend on their different properties. Additionally, different modification groups may endow hydrogel with different stimulus‐responsive proprieties.

The material which used to form hydrogel matrix could be divided into synthetic material and natural material. The synthetic hydrogels, which represented by poly(acrylic acid) (PAA), polyacrylamide (PAAm) and their derivatives, polyvinyl alcohol (PVA), and polyphosphazene (PPZ), are mostly used in various industries.^[^ ^68^ ^]^ The natural hydrogels, which represented by chitosan (CS), poly(lactic acid) (PLA), dextran, guar gum (GG), collagen, and their derivatives, are environmentally friendly and also widely used for the investigation of stimuli‐responsive hydrogels.^[^ ^69^ ^]^ Besides these common materials formed hydrogels, there are many novel hydrogels which composed of biomolecules such as proteins and nucleic acids, have also attracted a great attention of scientists from all over the world.

In this section, we listed several commonly used natural and synthetic biomaterials for the preparation of stimulus‐responsive hydrogels and introduced their nature briefly. Their medical applications were also mentioned in this part. As for biomolecule‐formed hydrogels, we will not present them here but in specific research later.

### 3.1 Natural Materials for the Preparation of Stimulus‐Responsive Hydrogels

Natural biomaterials are ordinarily obtained from creatures like plants, animals, or microorganisms. Many of them have good biocompatibility and biodegradability because of their natural existence in human bodies or other organisms. There are two primary methods for the produce of natural biomaterials: i) extracted from biotissues or creatures directly and followed by further operation such as purifying and enzymatic hydrolysis. This method is commonly used for the preparation of alginate and chitosan; ii) taking advantage of life activity of specific microorganism, like fermentation. This method is usually used to produce dextran and hyaluronic acid. Besides, according to the type of constituent molecule, natural biomaterials which used to form stimulus‐responsive hydrogels could be toughly divided into protein biomaterials and polysaccharide biomaterials (**Table** **3**).

**Table 3:** Classification of natural materials for hydrogel preparation.

Category	Biomaterial
Protein	Gelatin, peptide
Polysaccharide	Chitosan, guar gum, alginate, hyaluronic acid, dextran, cellulose, pectin, agar

In this section, we summarized several widely used natural biomaterials in stimuli‐responsive hydrogel research and applications. Their chemical structural formulas are shown in **Table** **4**.

**Table 4:** Chemical structures of some natural biomaterials.

Agents	Structural formula
Chitosan	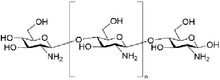
Hyaluronic acid	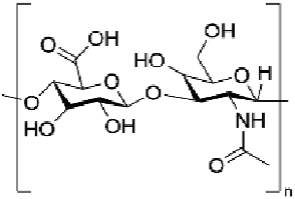
Sodium alginate	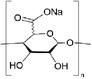
Dextran	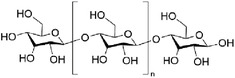
Cellulose	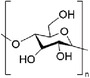
Gelatin	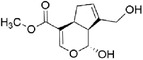
Guar gum	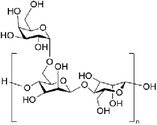
Peptide (e.g., Jelleine‐1)	e.g., PFKLSLHL‐NH_2_

#### 3.1.1 Chitosan and its Derivatives

Chitin is the only natural cationic polysaccharide, and abundantly exists in crustaceans and insects.^[^ ^70^ ^]^ While chitosan (CS) is the *N*‐deacetylated derivative of chitin with less toxicity, positive charged, and better mechanism property.^[^ ^71^ ^]^ Besides, CS can suppress the proliferation and growth of bacteria to a certain extent.^[^ ^72^ ^]^ Working as drug delivery, there are three important superiorities of CS. i) CS hydrogels can load a wide range of drug molecules, ranging from small molecules to supramolecular proteins. ii) After drug releasing, CS can be metabolized in the human body easily. iii) As said above, thanks to the wide distribution of chitin, the raw materials to prepare CS are cheap and easy to obtain.^[^ ^72^ ^]^ Because of the solubility difference at different pH, CS has been one of the most commonly materials to develop pH‐responsive drug release systems.^[^ ^73^ ^]^ Although CS‐based hydrogel possesses many merits for medical applications, the single administration of it is not effective enough for the treatment of inflammation or wound healing. Thus, to obtain functionalized CS hydrogel, physical and chemical cross‐linkings are mostly used.

**Figure 6:**
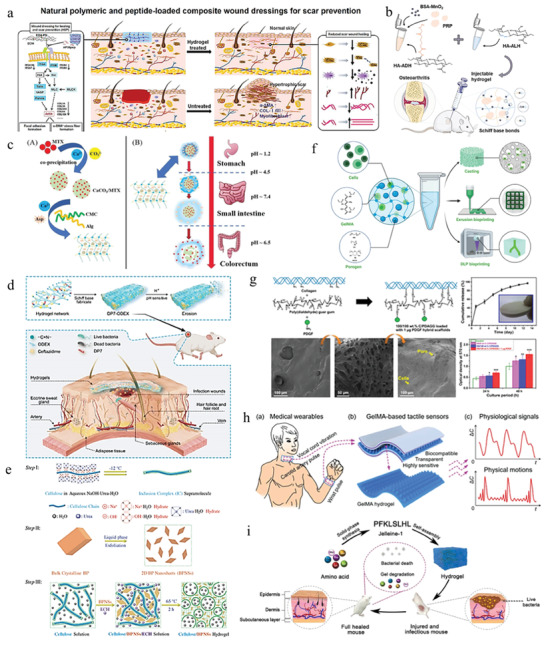
Natural materials‐based stimulus‐responsive hydrogels and their applications. a) A CS‐based wound dressing to prevent forming scar. Reproduced with permission.^[^ ^77^ ^]^ Copyright 2021, Elsevier B.V. b) A HA‐based and nanozyme‐composed injectable hydrogel for the alleviation of OA. Reproduced with permission.^[^ ^189^ ^]^ Copyright 2022, Elsevier. c) A pH‐responsive dual drug delivery hydrogel system made by alginate and cellulose for cancer therapy. Reproduced with permission.^[^ ^95^ ^]^ Copyright 2021, Elsevier. d) A dextran‐based antibacterial hydrogel dressing to accelerate wound healing. Reproduced with permission.^[^ ^105^ ^b]^ Copyright 2022, Elsevier. e) A cellulose and black phosphorus composed hydrogel for the photothermal therapy of cancer. Reproduced with permission.^[^ ^116^ ^]^ Copyright 2017, John Wiley & Sons. f) The application of GelMA for 3D bioprinting. Reproduced with permission.^[^ ^124^ ^]^ Copyright 2022, John Wiley & Sons. g) A GelMA‐based wearable biosensors used for the monitor of physiological and motion signals of human. Reproduced with permission.^[^ ^126^ ^]^ Copyright 2020, John Wiley & Sons. h) A collagen and guar gum‐based scaffold applied for tissue engineering. Reproduced with permission.^[^ ^130^ ^]^ Copyright 2022, Elsevier. i) A novel peptide‐based hydrogel for immunoregulation and enhanced radiation therapy effect. Reproduced with permission.^[^ ^134^ ^]^ Copyright 2022, Elsevier. B.V.

Theoretically, agents that can react or link with the reactive amino group of CS can be applied for the on‐demand modification of CS but those agents with high toxicity should be avoided. While acylation, alkylation, and carboxylation are regularly chemical methods for the preparation of CS‐based stimuli‐responsive hydrogels.^[^ ^72^ ^]^ Such as carboxymethyl chitosan (CMCS), which was found to be in favor of wound healing by promoting the repair of normal skin while inhibiting the proliferation of keloid fibroblast.^[^ ^74^ ^]^ A CMCS‐based injectable multifunctional hydrogel was reported by Jiang's group. They incorporated zinc oxide nanorods into CMCS to endow this hydrogel system for better antibacterial properties and then injected it into the irregular‐shaped wound. Finally, significant reduction of inflammation and wound healing promotion was observed.^[^ ^75^ ^]^ Recently, genipin has become an excellent natural cross‐linking agent due to the low toxicity and slow degradation rate.^[^ ^76^ ^]^ Wang and co‐workers investigated a genipin cross‐linked hydrogel networks which consists of CMCS, poly‐γ ‐glutamic acid, and antifibrotic polypeptide as an innovative wound dressing. Besides good biocompatibility and healing promotion, it can selectively inhibit the proliferation, migration, and activity of profibrotic cell phenotypes (**Figure** **6**a).^[^ ^77^ ^]^ Haeri and co‐workers developed thiolated chitosan‐based hydrogels for the mucoadhesive crocin delivery of crocin‐loaded niosomes for aphthous stomatitis.^[^ ^78^ ^]^ The sustained drug release achieved by the stimuli‐responsive hydrogel resulted in a prolonged effective period.

Physical cross‐linking mainly utilizes the linkage between different molecules or ions, such as hydrogen bonding and anion–cation interaction.^[^ ^79^ ^]^ Because of that the uses of toxic agents are avoided, the obtained hydrogels may have less toxicity and better bioavailability. Nonetheless, the applications of physical modified CS‐based hydrogels sometimes limited by its mechanical strength.^[^ ^72^ ^]^ Guo and co‐workers proposed a dual bond involving stimuli‐responsive hydrogel to deal with skin incisions and infected full‐thickness skin wounds. One is pH‐sensitive bond (catechol–Fe) and another is dynamic Schiff base bonds, they endow the hydrogel with properties of self‐healing and on‐demand dissolution or degradation.^[^ ^80^ ^]^

However, the clinical transformation of CS‐based stimuli‐responsive hydrogels is also limited by three major factors: i) debatable biocompatibility, ii) low mechanical properties and low water solubility, and iii) the raw materials of CS production mainly extracted from crustaceans. Therefore, some properties of CS, like molecular‐weight, maybe tremendously different depends on the difference of the species, climate, and environment of the habitats. These could be a hardship for the quality control of CS‐based products.

#### 3.1.2 Hyaluronic Acid and its Derivatives

Similar to alginate, HA is also a natural polysaccharide with good biocompatibility and widespread raw material source.^[^ ^81^ ^]^ But unlike the above kinds of stimuli‐responsive hydrogel materials, HA is a crucial ingredient to make up the human body and indispensable for organ constitution (e.g., skin, synovial fluid, vitreous body) and tissue repairing.^[^ ^82^ ^]^ As the development of biotechnology and gene engineering, nowadays HA is mainly produced by microbial fermentation instead of direct extraction from animal tissues, which is the precondition of the wide use of HA.^[^ ^83^ ^]^ In recent years, HA has become a frequently‐used hydrogel biomaterial with abundant products in the medical area such as HA‐based dermal filler, eye drops, and viscoelastic agent for ocular surgery.^[^ ^84^ ^]^

Since HA is a crucial component in tissue or organs like eyes and joints, it has attracted a lot of research in drug carriers, tissue engineering, and wound dressing.^[^ ^30^, ^85^ ^]^ In recent past, most works are aiming at combining HA‐based hydrogel with diverse agents, like anti‐inflammatory drugs,^[^ ^86^ ^]^ nucleic acid or cells,^[^ ^87^ ^]^ to obtain multifunctional HA‐based stimuli‐responsive hydrogel with enhanced therapeutic effective and minor side effects. For example, an HA/platelet‐rich plasma hydrogel was proposed by Gao and colleagues. By encapsulating MnO_2_ (a nanozyme which could catalyze the dissociation of ROS), the stimuli‐responsive hydrogels could retain longer in joints, promote cartilage repair, and relieve the inflammatory response, thus suppressing the further development of OA (Figure 6b).^[^ ^88^ ^]^ Tang and co‐workers investigated a poly(lactic‐*co*‐glycolic acid) (PLGA) nanoparticles (NPs)‐embedded HA‐based hydrogel film to achieve local and sustained genetic drug delivery. The engineered miRNA plasmid could effectively reduce the expression of COX‐1 and COX‐2, which play an important role in inflammatory response. Therefore, tendinitis in injured part could be controlled and finally results in the reduction of postoperative tendon adhesion.^[^ ^89^ ^]^ Zhou and co‐workers developed a FeSO_4_‐loaded hyaluronic acid hydrogel to treat bacterial keratitis. The Fe^2+^ was released after the injection of hydrogel and then induced the ferroptosis of *S. aureus* to realize the curation of keratitis.^[^ ^90^ ^]^

However, there are still some limitations about the application of HA, which need to be resolved. For instance, excessive hydrophilicity and insufficient stability, though these downsides could be improved by physical or chemical modifications.^[^ ^85^ ^c^ ^91^ ^]^ Besides, contrary to the foregoing, some researchers support that HA may boost the metastasis of cancer cells or the aggravation of inflammation.^[^ ^92^ ^]^ Though it has been verified that the promotion or depression for tumor depends on the molecular weight of HA, no agreement has been achieved about the critical value.^[^ ^93^ ^]^ Thus, more efforts should be made to avoid the conflicts, while maximize the unique merits of HA in the study of HA‐based stimuli‐responsive hydrogels.

#### 3.1.3 Alginate and Its Derivatives

Alginates (ALG) are extracted from brown algae,^[^ ^94^ ^]^ and generally exist as salt of cations such as sodium (Na^+^), barium (Ba^2+^), magnesium (Mg^2+^). While sodium alginate (SA) is the most commonly used alginate agent in various fields (Figure 6c).^[^ ^95^ ^]^ Excepting favorable properties like biocompatibility, mucoadhesiveness, and degradability,^[^ ^94^ ^]^ its pH‐sensitivity makes SA‐based hydrogel play a non‐negligible part in the research of drug release system.^[^ ^96^ ^]^ The mechanism of its pH response nature is described as follows. A mass of carboxylate radicals (─COO^−^) exhibit polyanion behavior in aqueous solution. Under acidic conditions, the ─COO^−^ interacts with H^+^ and transforms to carboxyl group (─COOH). These lead to the weakness of hydrophobicity together with contraction of molecule chains, which result in the shrinkage of hydrogel. As the pH value increasing, ─COOH disassociate gradually and finally cause the swell of hydrogel because of the repulsive force between ─COO^−^. Then drug molecules are released. Kong's group designed a pH‐sensitive SA–cellulose‐based hydrogel to achieve the dual administration of methotrexate (MTX) and aspirin (Asp). In their research, according to the difference of internal environment pH between the stomach, small intestine, and colorectum, this hydrogel exhibits disparate drug release behavior: in the stomach (pH ≈1.2), due to the shrinkage of molecular chains, this hydrogel could prevent the undesirable leakage of MTX and Asp; about 2 h after oral administration, the release of Asp is significantly facilitated in small intestine (pH ≈7.4) while MTX is mainly released in the colorectum (pH ≈6.5). Therefore, this pH‐responsive hydrogel realized the chemical therapy and pain relief simultaneously.^[^ ^95^ ^]^

#### 3.1.4 Dextran and Its Derivatives

Glucans are a glucose‐consisted high‐molecular‐weight polymer. According to the glycosidic bonds as being either α‐linked or β‐linked, it could be classified into α‐glucan (or dextran) and β‐glucan roughly.^[^ ^97^ ^]^ β‐glucans mainly exist in oats, fungus, algae, and bacteria.^[^ ^98^ ^]^ And it has been verified helpful in the regulation of blood glucose,^[^ ^99^ ^]^ metabolism of cholesterol,^[^ ^100^ ^]^ suppression of tumors and enhancement of immunity.^[^ ^101^ ^]^ While dextran and its derivatives are usually used to form stimuli‐responsive hydrogels. Thus, we mainly introduced the properties and applications of dextran herein.

At nature condition, dextran is mainly produced extracellularly from sucrose by several lactic acid bacteria while its industrial production is ordinarily based on the fermentation process of *Leuconostoc mesenteroides*.^[^ ^102^ ^]^ Medically, dextran has been used as a substitute for blood plasma for nearly 80 years, which also demonstrates its excellent biocompatibility for clinical applications.^[^ ^103^ ^]^ Furthermore, dextran has abundant hydroxyl groups, which are favorable for the chemical or physical modification and cross‐linking. Hence, dextran and its derivatives are a prospective biomaterial for the development of stimuli‐responsive hydrogels. Many recent studies have developed a series of dextran‐based hydrogel drug release systems for various purposes like cancer therapy,^[^ ^104^ ^]^ insulin release, and antibacteria (Figure 6d).^[^ ^105^ ^]^ For instance, Solomevich et al. reported a nanohydrogel prepared by dextran phosphate for the treatment of cancer. The hydrogel they prepared is pH‐sensitive due to the presence of phosphate groups, which lead this hydrogel to lower swelling ratio in acidic environment but release contents in neutral environment.^[^ ^106^ ^]^

Although dextran has been applied in extensive industries such as food and medicine since its discovery in the 19th century,^[^ ^107^ ^]^ as a novel biomaterial, more intensive research and developments on stimuli‐responsive hydrogels are required.

#### 3.1.5 Cellulose and Its Derivatives

Cellulose is the most abundant polysaccharide on earth and is a linear polymer made up by glucose. According to the source of cellulose, it could be classified into bacteria cellulose (BC) and plant‐derived cellulose.^[^ ^108^ ^]^ There are numerous hydroxyls, carboxyl on the molecular chains of cellulose, which make this biomaterial easy to be cross‐linked or react with modification agents.^[^ ^109^ ^]^ Furthermore, the intermolecular hydrogen bonding leads cellulose‐based hydrogel to better stability and mechanical property.^[^ ^110^ ^]^ Above these, coupled with its good biodegradability and renewability,^[^ ^111^ ^]^ cellulose‐based stimuli‐responsive hydrogels have attracted a large attention from researchers. Recent studies mainly focused on its utilization for wound dressing, drug carriers, and scaffolds for tissue engineering.^[^ ^112^ ^]^

In order to make cellulose more suitable to form hydrogels, it is usually made into cellulose nanofibrils (CNFs) or cellulose nanocrystals (CNCs) to obtain more uniform particle size and higher specific surface.^[^ ^113^ ^]^ CNFs are semicrystalline with a high aspect ratio and flexibility while CNCs are highly crystalline with lower aspect ratio but more rigid.^[^ ^114^ ^]^ According to different demands, CNCs and CNFs could be used to form self‐assembly hydrogels or incorporated into various hydrogel matrix by specific cross‐linked strategy to realize desired effect.^[^ ^109^, ^115^ ^]^

For example, a black phosphorus (BP) loaded cellulose‐based stimuli‐responsive hydrogels were proposed by Zhang and co‐workers. This hydrogel showed favorable photothermal therapy effect for tumors due to the presence of BP. And owing to the cross‐link of cellulose, it also has good mechanical properties (Figure 6e).^[^ ^116^ ^]^ Moreover, Han and co‐workers improved the mechanical performance, thermal stability, and biodegradability of cellulose‐based hydrogels by incorporating CNCs. Consequently, this hydrogel could provide a suitable microenvironment for the adhesion and proliferation of cells and thereby promote the regeneration of injured tissues and organs.^[^ ^117^ ^]^ Xu and co‐workers investigate an amoxicillin‐loaded oral hydrogel carrier with good biocompatibility through hydroxypropyl methylcellulose. In their research, this hydrogel greatly prolongs the preservation time to 12 weeks without cold chain. And also improve the compliance of patients by covering unpleasant smell of amoxicillin which make this hydrogel could be used for pneumonia treatment of newborns.^[^ ^118^ ^]^

However, the clinical translation of cellulose‐based stimuli‐responsive hydrogels is limited by its mechanical strength and potential cytotoxicity. Xia's group has demonstrated that cellulose may induce inflammation or cell damage.^[^ ^119^ ^]^ Thus, the biosafety of cellulose‐based stimuli‐responsive hydrogels should be evaluated prudently before clinical test.

#### 3.1.6 Gelatin and Its Derivatives

Gelatin, which prepared by the hydrolyzation of collagen, is a kind of widespread protein in both human and animals.^[^ ^120^ ^]^ Benefiting from its ease of engineered, strong hydrophilicity, and low cost, gelatin is an outstanding material for the investigation of drug carriers.^[^ ^121^ ^]^ Meanwhile, gelatin is often combined with various groups or ions to form stimuli‐responsive hydrogels for realizing realize different functions like sustained drug release and stimuli response.

For example, gelatin methacryloyl (GelMA), a derivative of gelatin modified by photo‐cross‐linkable methacrylamide groups, is commonly used in the preparation of hydrogels. The synthesis procedure of GelMA usually involves methacrylic anhydride and gelatin.^[^ ^122^ ^]^ Not only GelMA maintains the advantages of gelatin such as biodegradability, high water‐holding capacity, and excellent film‐formation ability, but also improved biocompatibility, less immunogenicity, and less expensive. Due to the chemical reaction of methacrylamide groups upon the exposure of ultraviolet (UV), GelMA will transform from liquid to solid, namely, photocuring.^[^ ^123^ ^]^ By virtue of this property, this material can work as bioink for emerging 3D bioprinting (Figure 6f).^[^ ^124^ ^]^ Besides, nanomaterials‐combined GelMA, which are considered as the next‐generation therapeutic platforms for tissue repair, has been verified feasible and effective in regenerative medicine.^[^ ^125^ ^]^ Zhao and co‐workers developed a GelMA‐based tactile sensors for medical wearables to obtain physiological signals (Figure 6g).^[^ ^126^ ^]^ And modified by diverse groups or ions, GelMA could be designed as stimuli‐responsive (e.g., pH, ROS) drug release systems to treat inflammations or tumors.^[^ ^127^ ^]^

#### 3.1.7 Guar Gum and Its Derivatives

GG, which extracted from the endosperm of herbaceous plant Cyamopsis tetragonoloba, is a hydrophilic polysaccharide material for the fabrication of stimulus‐responsive hydrogels.^[^ ^128^ ^]^ And because of its low toxicity, environmentally friendly, cheaper cost, special physical and chemical properties, GG is also applied in various industries such as food production and mineral engineering.^[^ ^129^ ^]^ In the medical field, GG is primarily developed for drug carriers, biosensors, scaffold for tissue engineering (Figure 6h).^[^ ^130^ ^]^

By cross‐linking with another hydrogel (e.g., sodium alginate) as well as agents which are able to react with hydroxyl (─OH), GG‐based hydrogels could possess aggressive enhanced aqueous solubility, adjustable swelling ratio, and more favorable biocompatibility.^[^ ^129^ ^]^ In these ways, the deficiencies of GG like low dissolution rate and structural instability are able to be improved.^[^ ^131^ ^]^ Recently, Seeli and Prabaharan reported a pH‐sensitive hydrogel by cross‐linking poly(methacrylic acid) modified GG with ethylene glycol dimethacrylate. In the acidic pH, the release of drug molecules was limited by restricted swelling degree of hydrogel. But rapid drug release rate was observed in the alkaline environment because of the larger swelling volume. These results demonstrated that this stimulus‐responsive hydrogel is suitable to serve as an orally administrative drug carrier of hydrophobic drugs.^[^ ^132^ ^]^

#### 3.1.8 Peptide‐Based Stimulus‐Responsive Hydrogels

Peptide, usually oligopeptide, formed by dehydration and condensation of multiple amino groups, is also a material with great potential for hydrogel preparation. Peptide is a common biomolecule that widely exists in organisms and plays an indispensable role in physiological activity, which endows peptide with inherent biocompatibility, biodegradability, bioactivity, and biosafety. Moreover, noncovalent interaction like hydrogen bond, hydropathy property, π–π stacking, and electrostatic effect allow peptide to form order and various structures by self‐assembly and could be used to construct various nanostructures. Besides, the existence of carboxyl, amino, and amount side chains provides sufficient sites for further functionalization.^[^ ^109^ ^]^ These features all allow peptide‐based hydrogel have outstanding flexibility in both design and modification.

Due to these merits, peptide‐based hydrogel has been developed as wound dressing, drug carrier and applied for cell culture, tissue engineering, and 3D printing.^[^ ^111^ ^]^ Wang's group used a natural amphiphilic peptide, Jelleine‐1, to develop an injectable peptide‐based hydrogel for wound repair. Jelleine‐1, which has a wide antibacterial spectrum, was first discovered in royal jelly and its nanofiber could gelation through self‐assembly in PBS. Subsequently, they reported that the utilization of this Jelleine‐1‐based hydrogel effectively facilitated the healing process of burn wounds infected with *Methicillin Resistant S. aureus* in mice.^[^ ^133^ ^]^ Liu and co‐workers proposed Smac‐TLR7/8 hydrogel for immunoregulation in tumor microenvironment and. In their work, succinic acid was used as a linker to conjugate TLR7/8a, which is a toll‐like receptor agonist, with Smac N7 peptide, which could enhance the radiosensitivity of tumors. This oligopeptide was further connected with a self‐assembling peptide KEF9 to obtain the Smac‐TLR7/8 nanofibers through amidation reaction. Then these nanofibers could form hydrogel through self‐assembly in sodium chloride solution. After injection, Smac‐TLR7/8 hydrogel could induce the suppressed M2‐type tumor‐associated macrophages repolarize to activated M1‐type. Thus improve the effect of radiation therapy and immune therapy simultaneously (Figure 6i).^[^ ^134^ ^]^

### 3.2 Synthetic Materials for the Preparation of Stimulus‐Responsive Hydrogels

Synthetic biomaterials with desirable properties for medical research are developing rapidly. Most of them are obtained by the polymerization of monomers or forming copolymer (CP) (**Table** **5**). Compared with natural biomaterials, synthetic biomaterials generally have more stable physicochemical properties and flexible design to form stimulus‐responsive hydrogels, and their molecular weight could also be adjusted on‐demand. The design flexibility is largely benefit from the self‐assemble process of the supramolecular hydrogels, which is mainly through noncovalent interactions. And the noncovalent interactions include host–guest interactions, hydrogen bonding, 𝜋–𝜋 stacking, hydrophobic effect ionic interactions, and interactions involving biomolecule moieties. It is noteworthy that the self‐assembly and gelation process of supramolecular hydrogels usually depend on the cooperation of multiple noncovalent interactions.^[^ ^135^ ^]^ Besides, numerous derivatives of these synthetic biomaterials could be synthesized by modifying with various agents or cross‐linking with different polymers. In this section, we summarized the properties and applications of some common synthetic biomaterials briefly. Additionally, according to different scientific purposes, in order to prepare hydrogels with better performance and desired function, researchers usually prepared block copolymer by using natural and synthetic biomaterials. In Subsection 3.2.7, we introduced frequently used block copolymers in recent studies.

**Table 5:** Chemical structures of some synthetic biomaterials.

Agents	Structural formula
Poly(lactic acid) (PLA)	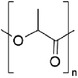
Polyacrylamide (PAAm)	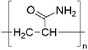
Poly(*N*‐isopropyl acrylamide) (pNiPAAm)	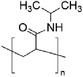
Poly(acrylic acid) (PAA)	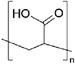
Poly(ethylene glycol) (PEG)	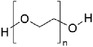
Polyvinyl alcohol (PVA)	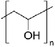
Poly(organophosphazene) (PNP)	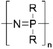
Poloxamer 407 (P407)/Pluronic F127	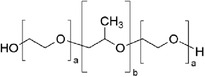

#### 3.2.1 Poly(Lactic Acid)

Poly(lactic acid) or polylactide (PLA) is a polyester produced by starch‐rich crops or vegetables such as corns and potatoes. As a promising biomaterial with nontoxicity, biodegradability, and desirable environmental performance,^[^ ^135^ ^]^ PLA has received more and more attention from researchers recently. Owing to its excellent degradability and biocompatibility, PLA is a regularly used material in the investigation of some medical consumables such as absorbable surgical sutures,^[^ ^136^ ^]^ bone fixation parts, and ocular implants.^[^ ^137^ ^]^ These consumables will dissolve gradually accompanied with the growth of damaged tissues. As a result, the re‐operations to remove scaffolds or sutures could be avoided, thereby enhancing the QOL of patients.^[^ ^138^ ^]^

**Figure 7:**
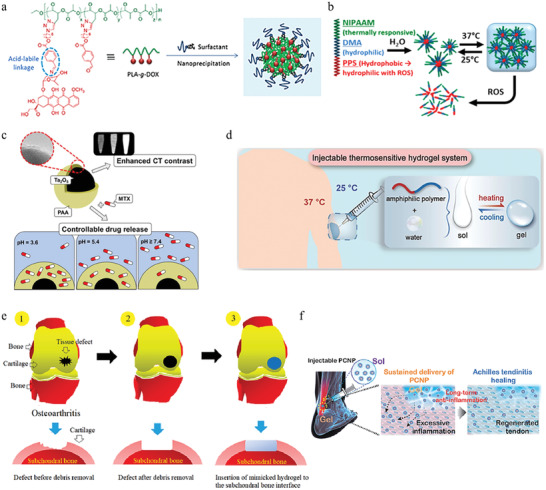
Synthetic materials‐based hydrogels and their applications. a) A PLA‐graft‐doxorubicin NPs used for precise drug delivery. Reproduced with permission.^[^ ^143^ ^a]^ Copyright 2014, American Chemical Society. b) A pNiPAAm‐based ROS/thermosensitive hydrogel for drug release. Reproduced with permission.^[^ ^144^ ^]^ Copyright 2014, American Chemical Society. c) A PAA‐based pH‐sensitive hydrogel used for controlled drug release. Reproduced with permission.^[^ ^145^ ^]^ Copyright 2019, Elsevier. d) A PEG‐based thermosensitive and biodegradable hydrogels. Reproduced with permission.^[^ ^146^ ^]^ Copyright 2022, Elsevier. e) A gelatin/PVA hydrogel used to mimic cartilage for the surgery of OA. Reproduced with permission.^[^ ^147^ ^]^ Copyright 2019, Elsevier B.V. f) An injectable celecoxib‐loaded PPZ‐based nanoparticle hydrogels for Achilles tendon regeneration. Reproduced with permission.^[^ ^148^ ^]^ Copyright 2019, Elsevier B.V.

Although PLA could not form hydrogel alone because of its hydrophobicity, it is widely used as the hydrophobic part to combine with some hydrophobic drugs in the block copolymer stimulus‐responsive hydrogels.^[^ ^139^ ^]^ Generally, hydrogels that composed of PLA could occur sol–gel transition at different temperature, namely, this material has good thermosensitivity.^[^ ^140^ ^]^ However, the downsides of PLA are also distinct and have become vital factors which impede its clinical transformation: i) poor toughness,^[^ ^141^ ^]^ ii) uncontrollable degradation rate,^[^ ^142^ ^]^ iii) difficult to be modified due to its linear structure and lack of active groups, and iv) hydrophobicity is a double‐edged sword while desirable in the design of drug carriers but unfavorable in the application of tissue engineering (**Figure** **7**a).^[^ ^143^ ^]^

In summary, PLA is still a potential emerging biomaterial but needs intensive research for its blending modification and action mechanism. With these, PLA‐based stimulus‐responsive hydrogels and medical consumables may become a boon to patients in the future.

#### 3.2.2 Polyacrylamide (PAAm) and Its Derivatives

Polyacrylamide is a linear high‐molecular polymer composed by acrylamide monomers and has high water‐holding capacity, chemical activity, and good thermostability.^[^ ^149^ ^]^ Besides, some properties of PAAm are able to be modulated as needed by adjusting the synthetic routes, forming copolymers with other monomers, chemical modification, or interacting with other hydrogels to form a cross‐linked network.^[^ ^150^ ^]^ As an important material, PAAm has been widely applied for the production of hemostatic plug, soft contact lenses, lubricant, and so on. Besides, PAAm and its derivatives‐based hydrogels have found research in several fields such as environmental protection and agriculture.^[^ ^151^ ^]^ For medical research, this nontoxic hydrogel with excellent swelling ratio and thermosensitivity has been developed as diverse smart drug release systems and biosensors.^[^ ^152^ ^]^

Due to various purposes and demands, numerous PAAm derivatives have been synthesized for the research of PAAm‐based hydrogels. For example, poly(*N*‐isopropyl acrylamide), which is a homopolymer of *N*‐isopropyl acrylamide, is also known as pNiPAAm. pNiPAAm is an amphiphilic biomaterial because of the simultaneous presence of hydrophobic isopropyl groups [(CH_3_)_2_CH─] and hydrophilic amide groups (─CONH_2_) in its monomer structure. As temperature increasing above the lower critical solution temperature (LCST), the hydrophobic interaction of isopropyl groups surpasses the hydrophilic interactions of amide groups, hence leading to the contraction of hydrogels.^[^ ^153^ ^]^ Namely, pNiPAAm is a favorable thermosensitive biomaterial. In addition, the LCST of pNiPAAm is ≈32 °C, which is similar to the human body temperature.^[^ ^154^ ^]^ Therefore, pNiPAAm is suitable for the development of thermosensitive drug release system. For example, Duvall and colleagues synthesized a thermoresponsive ABC triblock copolymer‐based hydrogel, which is composed of hydrophobic propylene sulfide (PPS), hydrophilic *N*,*N*‐dimethylacrylamide (PDMA), and pNiPAAm. The PPS‐*b*‐PDMA‐*b*‐pNiPAAm is micelles under room temperature (25 °C) but transform into more stable hydrogels under physiologic temperature (37 °C) (Figure 7b).^[^ ^144^ ^]^ Then, the ROS in tumor or inflammatory tissues could transform PPS from hydrophobic to hydrophilic. Finally, as the degradation of hydrogels, drug molecules are released into nidus.

However, slow response speed is a common obstacle which hinders the real applications of PAAm and its derivatives‐based hydrogels. Furthermore, there are also particular issues for specific PAAm derivatives. For instance, the clinical translation of the pNiPAAm‐based thermosensitive hydrogels is limited by its non‐biodegradability, though it has been verified nontoxic.

#### 3.2.3 Poly(Acrylic Acid)

PAA is a homopolymer synthesized by the polymerization of acrylic acid monomers, which has excellent water‐holding capacity, solubility, optical properties, and better biodegradability compared with pNiPAAm. The research and applications of PAA mainly utilized the abundant presence of carboxyl groups (─COOH) in acrylic acid monomers. For instance, by reason of the large existence of carboxylate (─COO─) when dissolved in water, this hydrogel could exhibit favorable activity to absorb heavy metalions, which could be applied for the environmental management.^[^ ^155^ ^]^ Besides, PAA appears weak acidity and excellent pH‐sensitivity due to the ionizable protons. Thus, biosensors and pH‐responsive drug release systems investigated by using PAA‐based hydrogels were also reported in recent years (Figure 7c).^[^ ^145^, ^156^ ^]^ Moreover, plenty of carboxyl groups make for the ease of its modification and grafting, in which varied and multifunctional PAA composed block copolymers are prepared for various medical applications.^[^ ^157^ ^]^

Ordinarily, PAA‐based hydrogels get contracted in acidic environment but rapidly swell under alkaline conditions. In light of this, Tsai and co‐workers developed a pH‐responsive hydrogel consist of chondroitin sulfate, and alginate‐graft‐poly(acrylic acid). This hydrogel was found to contract at pH 1.2 while have bigger volume at pH 7.4. In the consequent experiments, they further demonstrated that this hydrogel is appropriate for drug oral delivery.^[^ ^156^ ^b]^ Besides, Tey and co‐workers proposed a hepatitis B core antigen (HBcAg)‐sensitive hydrogel biosensing through immobilized HBcAg and the anti‐hepatitis B core antigen (anti‐HBc) antibody on PAA. This hydrogel biosensing possesses reversibility and reusability, and also has high sensitivity for HBcAg, which all make this device potential for hepatitis B detection.^[^ ^158^ ^]^

#### 3.2.4 Poly(Ethylene Glycol) (PEG) and Its Derivatives

As one of the most frequently used synthetic biomaterials, PEG, also known as polyethylene oxide (PEO) or polyoxyethylene, plays a crucial part in biomedical engineering. Accompanied with hydrophilicity and nonimmunogenicity, PEG is often used for surface modification in nanoparticles which could improve their biocompatibility greatly and realize long‐term internal circulation.^[^ ^159^ ^]^ As the resistance to protein absorption, PEG‐based hydrogels are also widely used in bone tissue engineering.^[^ ^160^ ^]^ Besides, PEG has been demonstrated have the capacity to induce the proliferation and differentiation of cells. Thus, many researchers are dedicating their efforts to explore the promising applications of PEG‐based hydrogels for extracellular matrix.^[^ ^161^ ^]^ For drug delivery and controlled release system, though PEG polymer could form hydrogel alone, in most cases, it is used to combine with some hydrophobic polymers to develop block copolymers to get required stimuli‐responsive hydrogels (Figure 7d).^[^ ^146^ ^]^

PLGA–PEG–PLGA triblock copolymer is one of the most widely studied PEG‐based biomaterials for the development of thermo responsive hydrogels.^[^ ^162^ ^]^ To improve the stability of PLGA–PEG–PLGA‐based hydrogel, Hennink's group prepared a PCLA–PEG–PCLA‐based hydrogel by replacing PLGA block with poly(ε‐caprolactone‐*co*‐lactide) (PCLA).^[^ ^163^ ^]^ This hydrogel is sol state at room temperature but will transform into a gel system at 37 °C. In vivo experiment, they indicated that this hydrogel could prolong the sustained release of drug molecules to 4 to 8 weeks. By loading celecoxib, a nonsteroidal anti‐inflammatory drug, this hydrogel could be a promising drug delivery platform for articular pain management.

#### 3.2.5 Polyvinyl Alcohol (PVA)

PVA is a familiar chemical or medical material, which could be found easily in the production of glue, cosmetics, and artificial kidney membrane. PVA is a hydrophilic polymer, and has a lot of advantages which are common to biomaterials, such as biocompatibility, biodegradability, and nontoxic.^[^ ^164^ ^]^ Nonetheless, the mechanical property of pure PVA hydrogel could become unstable under swelling state, like the deterioration of elasticity and hardness. Therefore, PVA is hardly used to form hydrogels alone but act as a modification agent or cross‐linker to form a composite hydrogel. Or improving the property of pure PVA hydrogels by doping metal ions.^[^ ^165^ ^]^ The most distinct characteristic of PVA‐modified hydrogels is the improved mechanical property compared with the simple hydrogels. Due to the cross‐linking hydrogen bonds between molecules, PVA‐modified hydrogels possess preferred toughness and strength.^[^ ^147^, ^166^ ^]^ By reason of this, PVA‐based hydrogels are commonly used in the investigation of vascular scaffold, wound dressing, and cartilage substitutes.^[^ ^167^ ^]^

Though stimuli‐responsive hydrogels are promising for the treatment of cancer or inflammation, sometimes they are limited by the weak mechanical strength which could lead to serious drug leakage or implant loosening. As mentioned above, PVA could be used as a modification agent, which could endow these hydrogels with desirable mechanical properties. For instance, Qi and co‐workers prepared a thermosensitive chitosan‐based hydrogel modified by glutaraldehyde and PVA. This hydrogel could achieve sol–gel transition at about 36 °C and have sufficient toughness. More importantly, this hydrogel system could avoid the burst release of PTX, namely, it has a flatter release curve and a longer drug release period.^[^ ^166^ ^]^ A thermoresponsive hybrid hydrogel cross‐linked by gelatin and PVA was carried out by Meesane and co‐workers. Subsequently, increased Young's Modulus and stress are observed in the mechanical tests (Figure 7e).^[^ ^147^ ^]^

PVA‐based stimuli‐responsive hydrogels could also be applied for extracellular matrix for cell culture because of the ability of cell adhesion. Scientists have verified that PVA could be able to work as a low‐cost but effective hematopoietic stem cell culture fluid, which could be promising for the treatment of leukemia.^[^ ^168^ ^]^

#### 3.2.6 Poluphosphazene (PPZ) and Poly(organophosphazene) (PNP)

PPZ is an inorganic polymer with a backbone formed by alternant nitrogen and phosphorus. It has both linear structures and ring structures. PPZ molecule has active chlorine atoms that are easy to replace by various side groups. By this, PPZ derivatives could be endowed with different properties like hydrophilicity or hydrophobicity, which also makes PPZ a promising material with outstanding design flexibility as the stimuli‐responsive hydrogels to be applied in multiple areas.^[^ ^169^ ^]^ While PPZ will become PNP when the chlorine atoms are replaced by organic side groups such as amino acids and vitamins. As a class of biomaterial which attracted great interest in medical research, PPZ and PNP have several non‐negligible advantages. First, excellent biodegradability and biocompatibility. PPZ and PNP usually degrade into harmless small biological molecules such as phosphoric acid and ammonia, and hardly trigger inflammatory response, which induced by immunological rejection. Inspired by this, they are frequently applied for absorbable surgical sutures and short‐term medical implants to avoid re‐operations.^[^ ^170^ ^]^ Besides, due to its insolubility and controllable glass‐transition temperature, they are also desired materials to form elastomers and stimuli‐responsive hydrogels.^[^ ^171^ ^]^

For the investigation of drug‐loaded hydrogels and tissue engineering, PNP‐based stimuli‐responsive hydrogels are more commonly used because of better biocompatibility and more diverse functions compared with PPZ.^[^ ^172^ ^]^ Ambrosio and co‐workers prepared a pH‐responsive PNP‐based hydrogel by modifying PPZ with methoxyethoxyethoxy side groups. This hydrogel shows a lower swelling ratio in acidic environment, which could be used for oral drug delivery platforms.^[^ ^173^ ^]^ Besides, Song's group, in order to treat tendonitis effectively and prolong the period of drug release, developed a local injectable thermoresponsive hydrogel system composed of PNP (aminoethanol PPZ) and celecoxib (CXB) loaded nanoparticles. The amphiphilic PNP‐based stimuli‐responsive hydrogel matrix also makes this system occur sol–gel transition under body temperature. Except for alleviating excessive inflammatory response, this hydrogel system also exhibited the ability to induce tissue regeneration by providing long‐term anti‐inflammatory effects (Figure 7f).^[^ ^148^ ^]^

At present, the real applications of PPZ‐ and PNP‐based stimuli‐responsive hydrogels are mainly constrained by its industrial production capacity. Particularly the production of hexachlorocyclotriphosphazene (N_3_P_3_Cl_6_), a raw material to produce PPZ, has gained no breakthrough for a long period. Maybe in the future, with the improvement of PPZ production process, this stimuli‐responsive hydrogel could be better used for medicine research.

#### 3.2.7 Block Copolymer Based Hydrogels

Block copolymer is a class of polymer material prepared by linking at least two different chain‐segments together. It could be classified into diblock, triblock, and multiblock copolymer based on the number of chain‐segment. Numerous effective and efficient methods have been developed to obtain block copolymers to form various stimuli‐responsive hydrogels.^[^ ^174^ ^]^ Because of the morphology and marshalling sequence of chain‐segment, block copolymers have unique properties, which are different from the simple mixture and graft copolymer with the same constituent. Coupled with the unparalleled design flexibility, their preparation, investigation, and applications have been a frontier, which was paid more and more attention by scientists. For the research of drug delivery platform, based on diverse demands, block copolymer could be made hydrophilic, hydrophobic or amphiphilic to form various stimuli‐responsive hydrogel systems, dendrimers nanospheres or nanocapsules.^[^ ^175^ ^]^

Poloxamer 407 (P407), also known as Pluronic F127, is a widely used amphiphilic ABA‐type triblock copolymer consisting of a hydrophobic poly(propylene glycol) (PPG) unit and two hydrophilic PEG units, namely, PEG–PPG–PEG (or PEO–PPO–PEO). Besides, P407 has thermosensitivity and good compatibility with many drug molecules and biomolecules.^[^ ^176^ ^]^ However, though P407 is an ideal raw material for the design of smart hydrogels, there are some concerns about its biosafety.^[^ ^177^ ^]^ Therefore, P407 is usually further modified for research and real applications. Kohane et al. designed a novel pentablock copolymer P407‐polybutylphosphoester (P407‐PBP) based thermoresponsive hydrogel system for the sustained release of ciprofloxacin, to realize radical cures of otitis media. In their research, enhanced hydrophobicity and thermosensitivity were observed after decorating the ends of P407 with aliphatic polyphosphoester and the side chains with butyl. Besides, by using chemical permeation enhancers, both the mechanical strength and the drug transtympanic permeation capacity of this hydrogel system were improved. In the subsequent experiments, they demonstrated the preferred therapeutic effect and better biocompatibility of the stimuli‐responsive hydrogel system.^[^ ^178^ ^]^

Additionally, P407 formed hydrogel is not biodegradable and ordinarily dissolves within a few days, which will exert an adverse effect on the drug release. In light of these, Kim and co‐workers synthesized a P407 structure‐similar ABA‐type triblock copolymer by replacing the center unit PPG with biodegradable PLGA. The PEG–PLGA–PEG will form a core–shell structure in aqueous solution and further form a hydrophobic drug‐loaded stimuli‐responsive hydrogel, which could persist releasing drug molecules up to 2 months and have good biocompatibility simultaneously.^[^ ^140^ ^]^

## 4 Endogenous Factors Responsive Hydrogels for Inflammation Therapy

Various inflammatory mediators and cytokines are released and play an essential role during inflammatory response. As shown in Table 2, most of these mediators, such as ROS, NO, and H^+^, have been developed as a switch to trigger hydrogels. Endogenous factors responsive hydrogels could start working without external stimulations. This mechanism makes these hydrogels both convenient and efficient. In this subsection, endogenous factors responsive hydrogels are introduced, their advantages, and conflicts are also discussed.

### 4.1 pH‐Responsive Hydrogels

**Figure 8:**
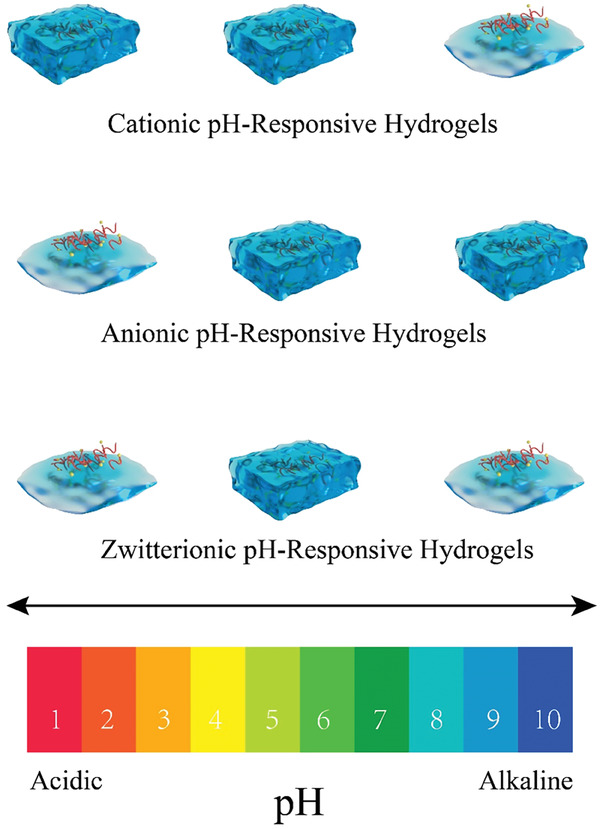
Classification of pH‐responsive hydrogels and their phase change behaviors at different pH.

The pH value in the human body varies greatly according to different tissues, organs, and even their physiological state (**Table** **6**), which provides opportunities for the development of targeted drug delivery. To date, pH‐responsive hydrogels are the most researched smart hydrogel system. Their main working principle is that the phase of these hydrogels could transform under different pH conditions. The pH threshold of phase change is affected by a lot of factors but mainly depends on the nature of side groups. Therefore, according to their different side groups, pH‐responsive hydrogels could be divided into cationic, anionic, and zwitterionic hydrogels (**Figure** **8**). In this section, we will introduce these three types of pH‐responsive hydrogel and their recent studies for inflammation therapy, respectively.

**Table 6:** The pH of various tissues/organs in the human body.

	Tissues/organs	pH	Refs.
Normal conditions	Stomach (gastric fluid)	≈1.2	[156]
	Intestine (intestinal fluid)	≈7.4	
	Blood and extracellular microenvironment	≈7.2–7.4	[183]
	Mouth cavity (saliva)	≈6.6–7.1	[184]
Pathological conditions	Solid tumor	≈6.5–6.8	[183]
	Endocytic vesicles of cancer cells	≈5.5–6.5	
	Inflamed joint	≈5–6	[181]
	Inflammatory colonic fluid	≈6.8	[185]
	Inflammatory sites	Usually ≈4–6, also depends on the type, inflammation phase, pathogens.	[186]

#### 4.1.1 Cationic pH‐Responsive Hydrogels

There are numerous cationic groups on the chains of polymers, which constitute the cationic hydrogels. Particularly, amino (─NH_2_) and imine (═NH) group are the most common type.^[^ ^179^ ^]^ Because of the protonation of these cationic groups, this kind of hydrogel will swell in an acidic environment and their swelling ratio will rise with the increase of the number of cationic groups. Therefore, the cationic hydrogels are mainly used for the development of injectable hydrogels and stomach drug delivery systems.

Aycan and Alemdar developed a chitosan‐based hydrogel for amoxicillin gastric administration.^[^ ^180^ ^]^ They cross‐linked chitosan‐grafted‐glycidyl methacrylate (CTS‐*g*‐GMA) with poly(ethylene glycol) diacrylate (PEGDA) to prepare a composite hydrogel network. Then, natural bone ash (BA) is added as a filler, which could endow this hydrogel system better mechanical strength and biocompatibility. Noted as (CTS‐*g*‐GMA)/PEGDA/BA. Because of the protonation of abundant amino groups under acidic environment, compared to pH 7.4, this hydrogel system showed higher swelling ratio at pH 1.2. Namely, (CTS‐*g*‐GMA)/PEGDA/BA could realize controlled amoxicillin release in gastric environment. Thus, it could improve the bioavailability of drugs and may be a promising material for gastric ulcer therapy.

Bioinspired by mussel, Zhang and co‐workers developed an injectable hydrogel system with enhanced lubrication and controllable drug release.^[^ ^127^ ^b]^ They decorated the GelMA hydrogel microspheres with dopamine methacrylamide–methacryloxyethyl phosphorylcholine (DMA–MPC) diblock copolymer. Then an anti‐inflammatory drug, diclofenac sodium (DS), was encapsulated in this hydrogel system. The GelMA@DMA–MPC@DS hydrogel system combined the biomimetic lubrication coating and drug delivery vehicle together. It could realize the pain relief and reduce the further degradation of cartilage synchronously.

To obtain an injectable hydrogel for the treatment of arthritis, Mi and co‐workers blended carboxymethyl hexanoyl chitosan (CHC) with low molecular weight hyaluronic acid (LMW HA) simply.^[^ ^181^ ^]^ On the one hand, because of the amphiphilic nature of CHC, LMW HA could act as a cross‐linker which could transform the blends into hydrogels. On the other hand, the electrostatic interactions between positively charged CHC and negatively charged LMW HA also contribute to the form of hydrogel. Similarly, a positively charged anti‐inflammatory drug, berberine, was assembled into this system based on the electrostatic interactions. Under acidic conditions, the electrostatic interaction in this hydrogel will be weak because of the protonation of carboxylate groups, and then lead to quick release of drugs. As anticipated, researchers subsequently noted that the berberine release rate was more rapid at pH 5.0 than pH 7.4. While the microenvironment of inflamed joint ranges from pH 5 to 6. Therefore, arthritis therapy based on this blending hydrogel system is demonstrated feasible.

**Figure 9:**
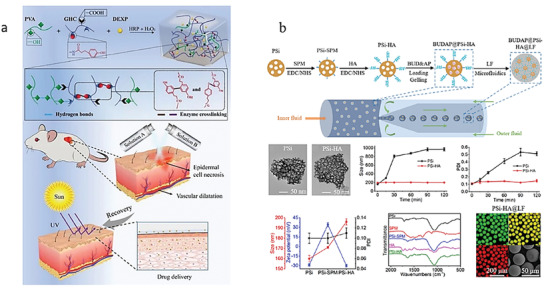
pH‐responsive hydrogels. a) A novel cationic pH‐responsive hydrogel‐based spray for the therapy of solar dermatitis. Reproduced with permission.^[^ ^182^ ^]^ Copyright 2022, Springer Nature Switzerland AG. b) Anionic pH‐responsive hydrogel‐based oral drug delivery system to inflamed sites in intestine. Reproduced with permission.^[^ ^87^ ^b]^ Copyright 2022, Elsevier.

Fan and co‐workers reported an enzyme‐catalyzed pH‐responsive semi‐interpenetrating polymer network hydrogel for solar dermatitis treatment.^[^ ^182^ ^]^ This hydrogel was prepared with gelatin, 3‐(4‐hydroxylphenyl) propionic acid (HPA), PVA, glycerol, and dexamethasone sodium phosphate (DEXP). First of all, HPA was grafted onto the gelatin by EDC‐NHS chemistry to obtain a GH conjugate (GHC). Then, Solution A consists of GHC and horseradish peroxidase (HRP), Solution B consists of PVA, glycerol, and H_2_O_2_ were prepared. The researchers mixed Solution A with B, attributing to the massive presence of carboxyl groups on GHC and hydroxyl groups on PVA and catalyzation of HRP and reaction of H_2_O_2_, a great deal of hydrogen bonds were generated and formed the network of hydrogel. The obtained GH/P‐1/DEXP‐2 hydrogel also has excellent capacity that prevents solar dermatitis from further deterioration by UV resisting because of the existence of glycerol and phenol groups. Moreover, the hydrogen bonds were susceptible to be destroyed under acidic environment, thereby sustained transdermal drug release could be achieved on the inflamed skin. By making Solution A and B into an agent spray, respectively, this multifunctional hydrogel is convenient to be used, which provides a promising strategy for solar dermatitis clinical therapy (**Figure** **9**a).

#### 4.1.2 Anionic pH‐Responsive Hydrogels

Anionic hydrogels swell at a higher pH but contract at a lower pH due to the presence of acidic groups on its polymer chains. The ionization of these groups, which is represented by carboxyl (─COOH) and hydroxyl (─OH), usually leads to the abundant formation of negatively charged side groups. Then, these side groups will cause repulsion and finally result in the swelling of anionic hydrogels. This kind of hydrogel is mainly used for drug delivery, especially for intestine delivery.

For example, Han and co‐workers developed a hydrogel system for the simultaneous delivery of siCD98 and curcumin (CUR).^[^ ^187^ ^]^ They first prepared a siCD98/CUR coloaded nanoparticle composed of PLGA and CS. After the reaction with HA solution, the HA‐functionalized NPs were obtained. Then, these NPs were entrapped in a hydrogel matrix based on chitosan and alginate. The mixed hydrogel matrix will collapse at colonic pH. The NPs are released subsequently. Due to the HA coated on NPs, they could target and uptake in macrophages by HA‐mediated receptor. Finally, the combination therapy for ulcerative colitis has come true.

Xu's group synthesized a nanocomposite drug for target therapy of intestine inflammation by reducing the TNF‐α level. The mainly effective constituent is phosphorothioated antisense oligodeoxyribonucleotide of TNF‐α (PS‐ATNF‐α). Its targeting capacity for the intestinal inflammatory cells mainly comes from the interaction of the single chain of triple helical β‐glucan (s‐LNT) with polydeoxyadenylic acid [poly(dA)], both of them are linked with PS‐ATNF‐α by disulfide bond. In order to deliver the s‐LNT/poly(dA)–SS–PS‐ATNF‐α to inflammatory tissue effectively and precisely, they encapsulated these nanocomposites into chitosan–alginate (CA) hydrogel. After oral administration, this CA hydrogel will swell and release amounts of s‐LNT/poly(dA)–SS–PS‐ATNF‐α at pH 6.8, which is also close to the pH of inflammatory colonic fluid. Subsequently, the decreased level of some proinflammatory factors was observed, indicating this hydrogel system could alleviate intestine inflammation significantly.^[^ ^185^ ^]^

Santos and co‐workers proposed a composite hydrogel system with hierarchical structure for the oral drug delivery to the inflamed site of intestine.^[^ ^188^ ^]^ First, an enzyme‐response NPs (BUDAP@PSi‐HA) was prepared by porous silicon NPs, HA, and acorbyl pllmitate, which have enzyme‐cleavable bonds and could be used to encapsulate budesonide (BUD, a glucocorticoid for intestine inflammation therapy). In order to realize intestine drug release and endeavor to avoid the leakage in the stomach, these obtained NPs were further encapsulated in hydroxypropyl methylcellulose acetate succinate (HPMCAS), which could be divided into three types, LF, MF, and HF based on their solubility at different pH. The BUDAP@PSi‐HA@HPMCAS hydrogel system first dissolved at intestine due to the pH change. Then, the released BUDAP@PSi‐HA NPs located to the inflamed site by electrostatic interaction. And following, these NPs were disassembled by the overexpressed matrix metalloproteinases (MMPs) and esterase, thereby the drug molecules were released to against the inflammation (Figure 9b).

Interestingly, Li and co‐workers designed DEX‐loaded transformable NPs.^[^ ^189^ ^]^ These NPs could transform into hydrogels in an acidic microenvironment. Thus, achieve sustained drug release in inflammatory sites. Furthermore, these nanoparticles could also form a hydrogel barrier on the mucosa of the stomach, which protects the gastric environment from undesired side effects after oral administration.

#### 4.1.3 Zwitterionic pH‐Responsive Hydrogels

The molecular chains of zwitterionic pH‐responsive hydrogel contain both acidic‐ and alkaline‐responsive groups. Therefore, this type of hydrogel could undergo phase change in a wide range of pH. Generally, zwitterionic hydrogels swell largely at both higher and lower pH while have lower swelling ratio at intermediate pH. For inflammation therapy, amphoteric hydrogels are mainly used as drug delivery system.

Zhou and co‐workers prepared a zwitterionic hydrogel (BC–AA–CS) by copolymerizing bacteria cellulose (BC), acrylic acid (AA), and CS.^[^ ^190^ ^]^ Due to the abundant presence of carboxyl groups and amino groups, this hydrogel exhibited special pH‐responsive property. The minimum swelling ratio appeared at pH 3.5–5.0 while larger swelling volume was observed at both higher and lower pH. They further utilized this hydrogel to delivery naproxen, which is a frequently used hydrophobic anti‐inflammatory drug to treat RA and gout. By adjusting the proportion of these three ingredients, this hydrogel could realize a lower drug release amount in acidic environment compared with alkaline environment. Therefore, sustained‐release was achieved and the adverse effects on the stomach triggered by naproxen are also reduced.

### 4.2 ROS‐Responsive Hydrogels

As shown in Table 2, ROS, which is represented by H_2_O_2_ and O^2−^, is a kind of crucial signal molecules in signal transduction and metabolism of cells. It has been verified that ROS is closely related to many proinflammatory pathways such as NF‐κB.^[^ ^191^ ^]^ Besides, ROS possess outstanding anti‐microbial capacity, which could destroy cytoderm of microbe directly. Normally, under inflammatory conditions, ROS will also promote macrophages to phagocytize pathogens.^[^ ^192^ ^]^ However, overproduced ROS will lead to the oxidation of DNA, proteins, and lipids. These have been verified related to many diseases or tissue dysfunctions, such as cancer, chronic inflammations, atherosclerosis, and cell aging.^[^ ^193^ ^]^

According to the response pattern and working principle, this kind of hydrogels could be mainly classified into two types: i) ROS‐catalyzing inorganic NPs embedded hydrogels, and ii) ROS‐reacting organic groups composed hydrogels.

#### 4.2.1 ROS‐Catalyzing Inorganic NPs Embedded ROS‐Responsive Hydrogels

Several inorganic NPs with antioxidative effects have been demonstrated could serve as an enzyme mimetic to remove overproduced ROS.^[^ ^194^ ^]^ The most studied inorganic NPs with the ability for ROS catalyzing could be classified into two types roughly: i) polyvalent metal oxide NPs, such as iron oxide (represented by Fe_2_O_3_), manganese oxide (represented by MnO_2_), and cerium oxide (represented by CeO_2_) NPs; and ii) carbon NPs. However, these inorganic NPs are limited by some factors, such as instability in harsh conditions, poor pharmacokinetics, nonspecific tissue accumulation, and potential harmful side effects.^[^ ^2^ ^a]^ Therefore, various ROS‐catalyzing inorganic NPs embedded hydrogels were developed to overcome the above limitations. And generally, these hydrogel matrixes themselves are not able to response to the ROS, but provide a protection, stable release or physical support for NPs.

**Figure 10:**
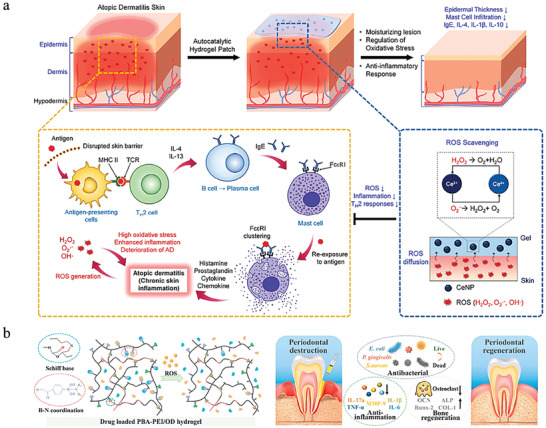
ROS‐responsive hydrogels. a) A CeO_2_ nanoparticles embedded alginate hydrogel patch to treat atopic dermatitis (AD). Reproduced with permission.^[^ ^58^ ^]^ Copyright 2022, Elsevier. b) An injectable hydrogel with ROS‐cleavable bonds to relieve chronic periodontitis. Reproduced with permission.^[^ ^199^ ^]^ Copyright 2021, John Wiley & Sons.

For example, Kim et al. carried out a CeO_2_ NPs embedded alginate hydrogel patch to treat atopic dermatitis (AD).^[^ ^58^ ^]^ According to the transformation between Ce^3+^ and Ce^4+^, abundant ROS are consumed while O_2_ and H_2_O are released. AD is a chronic inflammation mainly triggered by allergens, and has been verified closely related to high oxidative stress derived from ROS. After treating for the AD‐induced skin, this hydrogel patch exhibited excellent ability to relieve inflammatory response and promote wound healing (**Figure** **10**a).

Han and co‐workers investigated a multifunctional nanocomposite hydrogel system to repair osteoporotic bone defects. This hydrogel system is composed of methacrylated poly(glutamic acid)/GelMA matrix (m‐PGA/GelMA), and embedded with a novel microsphere, noted as FAPi‐MMS consisted of calcium phosphate (CaP), MnO_2_, dopamine, and fibroblast activating protein inhibitor (FAPi). These ingredients play different roles in the process of bone repairing: m‐PGA/GelMA endows this system with stable drug‐release rate due to its appropriate degrade rate; CaP provides a mesoporous scaffold for FAPi and MnO_2_; FAPi promotes the bone tissue regeneration by limiting the expression of FAP;^[^ ^194^ ^]^ MnO_2_ could consume H_2_O_2_ and produce O_2_, thereby rebalance the concentration of M1 and M2 macrophages. By ROS‐scavenging and immunoregulating, this nanocomposite hydrogel system finally realizes the relief of inflammation and promotion of bone tissue repairing simultaneously.^[^ ^195^ ^]^

Zhao's group reported a hydrogel carrier (PLE@MnCoO/Gel) to protect the injected bone marrow derived mesenchymal stem cells (BMSCs) from harsh IMEs of RA, thereby enhancing the therapeutic effects of stem cell transplantation.^[^ ^196^ ^]^ First, the ε‐polylysine (ε‐PLE) was grafted on the MnCoO NPs through electrostatic interaction to get a metal–organic NPs (ε‐PLE@MnCoO). Then, the ε‐PLE@MnCoO NPs were added in to the hydrazide‐modified HA (HA‐HYD) solution. Subsequently, the aldehyde‐modified HA (HA‐ALD) was added into the above mixture. The PLE@MnCoO/Gel hydrogel was obtained because of the formation of dynamic acylhydrazone bonds between ─CHO groups of HA‐ALD and ─CONH‐NH2 groups of HA‐HYD. The researchers further injected the PLE@MnCoO/Gel hydrogel into a 3D printed titanium alloy prosthesis scaffold (pTi@Gel‐NPs). The MnCoO NPs could catalyze the decomposition of H_2_O_2_ and generate O_2_, which is beneficial for the growth and differentiation of BMSCs. At the 12 weeks since implanting the pTi@Gel‐NPs into the diseased joint of RA rabbits, more significant suppression of inflammatory cytokines level and promotion of osseointegration were observed compared with pTi single treated and control groups.

#### 4.2.2 ROS‐Reacting Organic Groups Composed ROS‐Responsive Hydrogels

Besides the inorganic NPs mentioned above, there also many organic groups are exploited to form ROS‐responsive hydrogel system. The common feature of these groups is that all of them will occur structural change or bond break under ROS‐overproduced environment, namely, has reducibility. Boronic acid, sulfur‐based groups, polysaccharide, and phenol are the most common reagent types to modify or cross‐link with these hydrogels (**Table** **7**). Besides, there also a type of ROS‐reacting organic groups composed hydrogel could not react with ROS directly but response overproduced ROS by loading organic NPs, which is similar to the above ROS‐catalyzing inorganic NPs embedded hydrogels.

**Table 7:** Reaction scheme of common ROS‐reacting organic groups.

Organic groups	Reaction scheme
Boronic acid	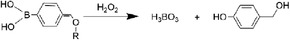
Phenylboronic ester	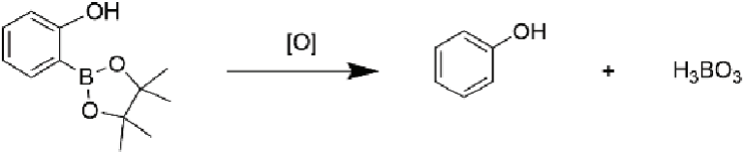
Thioether	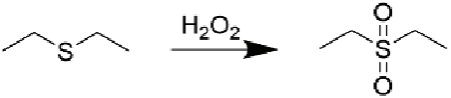
Thioketal	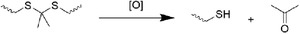
Disulfide bond	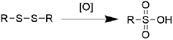
Glucosidic bond	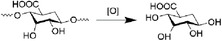

Recent years, probiotics have been proved effective for the therapy of colitis and enteritis. But the harsh environment of stomach leads to a largely death of these probiotics. To increase the survival rate of probiotics and enhance the therapeutic effect, Mu and co‐workers proposed a probiotics‐encapsulated ROS‐triggered hydrogel by cross‐linking methacrylate HA and thiolated thioketal.^[^ ^197^ ^]^ The number of living beneficial bacteria is significantly increased thanks to the protection of the hydrogel. Besides, the negatively charged hydrogel will adhere to the positively charged inflamed tissue due to the electrostatic interaction. Then, the thioketal linkage was cleaved by ROS, followed by the degradation of the hydrogel network and probiotic release. Because of the physiological activities of these probiotics, accompanied with the part elimination of excessive ROS caused by the break of thioketal bond, the inflammatory microenvironments are regulated, thus the further development of inflammation are controlled and a balanced microorganism environment is also reconstructed.

Chen and co‐workers proposed a novel hydrogel microsphere for the therapy of OA.^[^ ^198^ ^]^ They first prepared a triblock copolymer PEG–polycaprolactone (PCL)–N_1_‐(4‐boronobenzyl)‐N_3_‐(4‐boronobenzyl)‐N_1_,N_1_,N_3_,N_3_‐tetramethylpropane‐1,3‐ diaminium (TSPBA), noted as PEG–PCL–TSPBA. Together with DEX, Kartogenin (KGN), an inducer that could promote chondrogenic differentiation of stem cells and protect cartilage from further degeneration, is also loaded in the PEG–PCL–TSPBA nanoparticles. Moreover, WYRGRL, a peptide which could reinforce the cartilage‐targeting ability of the hydrogel microspheres by targeting the collagen II, is encapsulated in the GelMA together with the above NPs. Due to the presence of the arylboronic ester structure, these KGN/Dex‐TSPBA@WHM hydrogel microsphere will degrade in response to the excessive ROS. After intra‐articular injection, these hydrogel microspheres suppress the expression of proinflammatory cytokine significantly, which revealed the remarkable therapeutic effect for OA.

Li and co‐workers proposed an injectable hydrogel to relieve chronic periodontitis with diabetes mellitus (CPDM) efficiently.^[^ ^199^ ^]^ In their research, the phenylboronic acid‐functionalized poly(ethylene imine) (PBA‐PEI) was first synthesized by EDC‐NHS chemistry. Subsequently, the oxidized dextran (OD) was cross‐linked with PBA‐PEI by generating Schiff base. Furthermore, doxycycline (Doxy) which is an antibiotic and metformin (Met) and a hypoglycemic drug was coloaded into the hydrogel network by B–N coordination. When the drug‐loaded PBA‐PEI/OD hydrogels are injected into the diseased gingival tissue, the linkage between drug molecules and PBA are broken by the excessive ROS. Benefiting from the dual delivery of Doxy and Met and the ROS‐consume caused by hydrogel network, the undesired inflammatory response is considerably alleviated. Hence, this drug‐loaded PBA‐PEI/OD hydrogel shows excellent prospective for CPDM management (Figure 10b).

Yao and co‐workers prepared an HT/HGA hydrogel by combining antioxidant gallic acid‐grafted hyaluronic acid (HGA) with HA‐tyramine (HT) polymer through dual‐enzymatically cross‐linking method. This injectable hydrogel is biocompatible and possesses scavenging capacity against ROS. In their later experiments, decreased levels of TNF‐α and IL‐6 were observed after injection into brain, which verified the effectiveness of this hydrogel for neuroinflammation control and could be applied for traumatic brain injury repair.^[^ ^200^ ^]^

Wang and co‐workers reported an injectable hydrogel (Rapa@Gel) to alleviate intervertebral disk degeneration (IVDD).^[^ ^201^ ^]^ The ROS‐degradable hydrogel matrix was prepared by mixing an ROS‐labile linker, which has two kinds of phenylboronic acid with PVA. The hydrogel networks were collapsed in the ROS‐excessive microenvironment and the loaded rapamycin were released rapidly. Then, the drug molecules acted on immune cells to rebalance the concentrations of M1 and M2 macrophages at the inflamed tissues, thereby suppressing undesirable inflammation and promoting bone repair.

### 4.3 NO‐Responsive Hydrogels

As shown in Table 2, the properties of RNS are extremely similar to ROS, such as the strong oxidation and anti‐microbial ability. Additionally, a lot of physiological functions of ROS and RNS are also overlapped. Such as working as cellular signal molecules to regulate the proinflammatory pathways, modulating the expression of some proteins and enzymes, and even the indiscriminate attack on autogenous components when overproduced. Therefore, the strategies of RNS scavenging and design methods of RNS‐responsive hydrogel systems are also alike to which of ROS‐responsive hydrogels. Nitric oxide (NO), which is mainly generated from the enzymatic activity of NO synthase (NOS), is the most studied RNS and a great deal of achievements have been done. NO‐responsive hydrogel system usually incorporates a NO‐cleavable cross‐linker, such as o‐phenylenediamine. Herein, we will take NO‐responsive hydrogel as an example to summarize the working principle and recent progress of the RNS‐responsive hydrogels.

Numerous research has confirmed that excessive NO in tissues is associated with some tough diseases such as RA.^[^ ^202^ ^]^ In consideration of this, many NO‐responsive hydrogels related studies which focus on the therapy of RA have been carried out. For example, Kim et al. developed an injectable nanohydrogel (NO‐Scv gel) by polymerizing acrylamide and a NO‐cleavable cross‐linker (NOCCL, *N*,*N*‐(2‐amino‐1,4‐phenylene) diacrylamide) to deliver DEX for the treatment of RA. Because of the presence of o‐phenylenediamine group, the nanohydrogel network formed by NOCCL will degrade in a NO‐excessive environment. Hence, the NO‐Scv gel could remove NO and release drug molecules at a stable rate simultaneously.^[^ ^203^ ^]^

**Figure 11:**
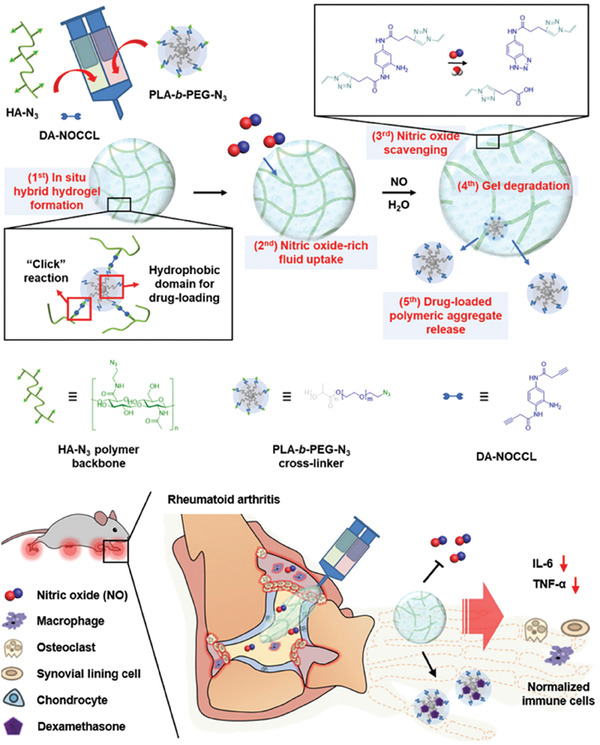
NO‐responsive hydrogel. The injectable M‐NO hydrogel system to realize the alleviation of RA by releasing DEX and savaging the excessive NO. Reproduced with permission.^[^ ^204^ ^]^ Copyright 2021 John Wiley & Sons.

Later, in 2021, Kim and co‐workers further improve their NO‐responsive RA therapeutic platforms by preparing a novel injectable self‐healing hydrogel platform named M‐NO to achieve NO scavenging and longer anti‐inflammatory drug release period at the same time.^[^ ^204^ ^]^ The M‐NO hydrogel is mainly composed of three parts: first, azide‐functionalized HA (HA‐N_3_) which endows this hydrogel system with good biocompatibility and mechanical property similar to synovial fluid; second, dialkyne‐functionalized NOCCL (DA‐NOCCL, *N*,*N*‐(2‐amino‐1,4‐phenylene) dipentyn‐4‐amide), which makes this system NO‐responsive; and third, azide‐functionalized PEG–PLA diblock copolymer (N_3_–PEG–PLA), which formed a hydrophobic area to load DEX. Since M‐NO hydrogels are injected into the RA‐induced articular, the superfluous NO leads the covalent interaction based on DA‐NOCCL to break. Accompanied with the degradation of hydrogel networks, DEX‐loaded polymeric aggregates are released gradually. And furthermore, DEX molecules are released to inflamed tissues by Fickian diffusion or erosion. Moreover, M‐NO hydrogel will reassociate through an entropy‐derived hydrophobic association when the concentration of NO decreased. Therefore, the capacities of on‐demand drug releasing and NO scavenging have been affirmed. Coupled with the alleviating effect of inflammations, M‐NO could be a promising candidate material for RA treatment (**Figure** **11**).

However, it is worth noting that the concentration of NO in vivo, even in inflammatory tissues, is quite low, which may limit the therapeutic effect of NO‐responsive hydrogels. To address this problem, Liang et al. proposed a nitrite (NO_2_^−^)‐responsive hydrogel as a drug carrier, and scavenge NO indirectly by modulating chemical equilibrium.^[^ ^181^ ^]^ Nitrite is the major metabolite of NO, which could be extremely harmful and toxic to human when overproduced.^[^ ^205^ ^]^ Besides, nitrite has higher concentration than NO in vivo, which makes it potential to act as a trigger of smart hydrogels. Inspired by this, a three‐arm dropyridine cross‐linker, DHPL, was prepared by researchers. The 4′ C─C bond of DHPL could be cleavage by oxidative aromatization so it possesses favorable capacity to response NO_2_^−^. Then, they cross‐linked the acrylamide (AAm) with DHPL to obtain the DHPL‐GEL. In order to enhance the anti‐inflammatory effect and realize sustained drug release, MTX liposomes were encapsulated into the hydrogel network. Eventually, in the inflammatory arthritis rat model, effective scavenge of NO, NO_2_^−^ and significant decreased inflammatory marker (e.g., TNF‐α, IL‐6) were observed, which indicate the alleviation of arthritis. Moreover, adverse side effects are also avoided.

### 4.4 Enzyme‐Responsive Hydrogels

It has been confirmed that several types of enzymes will be expressed and activated more in inflamed tissues than normal, such as ALPs, human neutrophil elastase (HNE), and MMPs.^[^ ^11^, ^12^, ^13^ ^]^ In recent years, inflammation therapy based on enzyme‐responsive hydrogels has gained much interest as its greener material source, stable drug release rate, and better selectivity. The most basic mechanism of this kind of hydrogels is that the excessive inflammation‐associated enzyme will break the cross‐linking bond in the networks, which is followed by the phase transition of hydrogels. Depending on the substance catalyzed by enzyme, the enzyme‐responsive hydrogels could be classified into four main categories: lipid‐, peptide‐, nucleic acid‐, and polysaccharide‐based hydrogels.

#### 4.4.1 Lipid‐Based Enzyme‐Responsive Hydrogels

Lipids with amphiphilic groups are suitable for the development of self‐assemble hydrogel system. Among them, triglycerol monostearate (TG‐18) formed hydrogels for the treatment of various inflammations are widely studied. TG‐18 has both a hydrophilic hydroxyl head group and a hydrophobic methylene group, which enable TG‐18 to form a self‐assembled structure in aqueous solution. Furthermore, a mass of hydrogen bonds formed intermolecular made TG‐18 could be used to prepare hydrogels with desirable mechanical property. Moreover, TG‐18‐based hydrogels will degrade in response to inflammatory environment due to the presence of ester bond which could be cleaved by MMPs and esterase.

**Figure 12:**
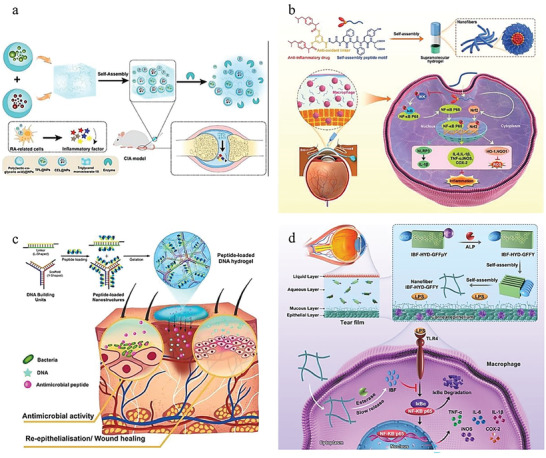
Enzyme‐responsive hydrogels. a) A novel injectable self‐assembly TG‐18‐based MMPs‐responsive hydrogel to treat inflammatory arthritis Reproduced with permission.^[^ ^206^ ^]^ Copyright 2021, Springer Nature Switzerland AG. b) A polyphenol‐based hydrogel eye drops to relieve the uveitis by regulating the macrophages. Reproduced with permission.^[^ ^209^ ^]^ Copyright 2022, John Wiley & Sons. c) ALPs‐responsive hydrogel eye drops for sustained‐release of IBF to treat uveitis. Reproduced with permission.^[^ ^55^ ^]^ Copyright 2022, Elsevier. d) Nuclease produced by bacteria to trigger the drug release of a novel DNA‐based hydrogel. Reproduced with permission.^[^ ^215^ ^]^ Copyright 2019, Elsevier.

For example, Karp and colleagues obtained an injectable self‐assembly TG‐18 hydrogel with triamcinolone acetonide (TA) loaded by heating these two agents in water.^[^ ^25^ ^a]^ Serious flares of inflammatory arthritis are mainly related to the overexpression of MMPs, thereby this hydrogel could achieve on‐demand drug release to inflamed site after intra‐articular injection. Similarly, Zhang and co‐workers investigated a TG‐18‐based hydrogel system for the treatment of RA.^[^ ^206^ ^]^ At the beginning, they synthesized the PLGA NPs as a drug vehicle to carry triptolide (TPL) and celastrol (CEL), both of which have satisfactory therapeutic effects for RA. And thanks to the encapsulation of PLGA NPs, CEL, and TPL could be released for a longer time, which prolong the duration of anti‐inflammatory effect. Subsequently, the drug‐loaded PLGA NPs were encapsulated into the TG‐18 formed hydrogel to gain the capacity of MMPs‐response. The remarkable relief of RA symptoms and significantly suppressed expression level of proinflammatory cytokines both revealed the good prospect of PLGA/TG‐18 hydrogels for the RA therapy (**Figure** **12**a). Additionally, Karuppannan and co‐workers utilized the injectable TG‐18 hydrogel for the delivery of zingerone.^[^ ^207^ ^]^ All of the above studies demonstrated the feasibility of TG‐18‐based enzyme‐responsive hydrogels for anti‐inflammation.

Moreover, Fu and co‐workers used TG‐18 hydrogel for the therapy of inflammation related eyelid diseases.^[^ ^208^ ^]^ The rosiglitazone (Rosi), which is an euglycemic agent with certain anti‐inflammatory effect, was loaded in the self‐assemble hydrogel network. In their consequent experiments, the Rosi‐loaded TG‐18 hydrogel was injected under the eyelid skin near the meibomian glands (MGs) area of mice model. The excessive MMPs that exist in the diseased MGs lead to the swell of hydrogels and lead to the drug release at a stable rate. The reduction of inflammatory symptoms was observed eventually. Li and co‐workers prepared a supramolecular filament hydrogel with polyphenol, IBF, and peptide motif to serve as an eye drop for the therapy of uveitis.^[^ ^209^ ^]^ This hydrogel was formed by the self‐assemble of abundant nanofiber units and could also convert back into nanofibers in inflamed sites and then penetrated into macrophages. Due to the catalyzation and hydrolyzation induced by esterase, the nanofibers were divided into antioxidative 3,5‐dihydroxybenzoic acid and anti‐inflammatory IBF to repress the inflammation and oxidative stress at the same time. Later, remarkable alleviation of uveitis was monitored in rabbit models (Figure 12b).

#### 4.4.2 Peptide‐Based Enzyme‐Responsive Hydrogels

Hydrogels formed by functionalized peptides with amphiphilicity are a potential inflammation therapy platform with much research by scientists. The peptide‐based enzyme‐responsive hydrogels will be disassembled due to the catalyzation of elastase or other proteases. Hence, as shown in Table 2, the activated HNF in the IMEs could be utilized to trigger the drug release of peptide‐based enzyme‐responsive hydrogels.

For instance, Matson and co‐workers carried out an elastase‐triggered hydrogel system to realize the sustained release of H_2_S.^[^ ^210^ ^]^ While the administration of H_2_S has been studied as a promising treatment for some chronic inflammations such as ulcers and chronic pneumonia. For a start, they cross‐linked carboxyl cellulose (CMC) with HNF‐degradable peptide‐aldehyde FBA‐VKVKVK (FBA = 4‐formylbenzamide). Later, to improve the biocompatibility, they replaced a certain percentage of CMC with carboxylic acid‐modified PEG (HOOC–PEG–COOH). Then, the peptide‐cross‐linked CMC–PEG hydrogel was reacted with S‐benzoylthiohydroxylamine and as a result the aldehyde groups were transformed into S‐aroylthiooxime (SATO) groups. The SATO group will release H_2_S when react with cysteine (Cys). While high level Cys usually means inflammations and thus, it is an important indicator for clinical diagnosis. In the simulative IMEs, the activated HNE triggered the gel–sol transition of CMC–PEG hydrogel, which leads to the exposure of SATO groups on the hydrogel surface and H_2_S was generated immediately. Furthermore, they also observed the protective effect of this hydrogel for cardiomyocytes by preventing the undesired side effects that triggered by doxorubicin, which reveal that the CMC–PEG hydrogel could be applied for the combination therapy of inflammations.

The researchers have noted that compared with a healthy person, the concentration of ALPs is higher in the tear fluid of anterior uveitis patients, though the exact pathogenesis of which is still unclear. In view of this, Li and co‐workers synthesized a dual‐enzyme‐responsive eye drops as the hydrogel precursor to achieve the sustained IBF released for the alleviation of anterior uveitis.^[^ ^55^ ^]^ They started by preparing a phosphorylated peptide‐drug (IBF‐HYD‐GFFpY) precursor. In the tear fluid, after the dephosphorylation induced by the overproduced ALPs, the IBF‐HYD‐GFFpY could be transformed into IBF‐HYD‐GFFY and subsequently formed a self‐assembly hydrogel system. Due to the existence of GFFY, the IBF‐HYD‐GFFY nanofibers were easily engulfed by activated macrophages. Later, the esterase in the cytoplasm of macrophages cleaved the ester bonds and lead to a slow release of IBF. Compared with conventional DS eye drops, this novel eye drops not only show a better therapeutic effect but also prolong the drug release period to 96 h (Figure 12c). Huang and co‐workers synthesized a PEG‐based hydrogel for intraoral drug delivery to treat periodontitis.^[^ ^211^ ^]^ The diacrylate‐modified PEG was linked by a Cys‐terminated peptide cross‐linker (CGPQG↓IWGQC), which could be cleaved by MMP‐8. And the MMP‐8 is a crucial proinflammatory enzyme that is present in the IMEs of periodontitis. This MMP‐8‐responsive hydrogel delivery vehicle appeared favorable drug release behaviors in the simulated inflammatory environments when loading different drug molecules.

#### 4.4.3 Nucleic Acid‐Based Enzyme‐Responsive Hydrogels

Nucleic acid‐based hydrogels, mainly represented by DNA‐based hydrogels, are a novel type of hydrogel with rapid development in recent decades. Compared with conventional hydrogels, DNA‐based hydrogels not only have good biosafety, biocompatibility, and biodegradability, but also possess excellent ability for specific molecular recognition and better design flexibility, which is reflected in its multifunctionality and programmability.^[^ ^212^ ^]^ Thus, DNA‐based hydrogels are widely studied in numerous fields such as biosensors, cancer research, and inflammation treatment.^[^ ^213^ ^]^ Herein, we will introduce two examples of inflammation management through DNA‐based enzyme‐responsive hydrogels.

It has been confirmed that the bacteria which infect the human body will produce abundant detrimental factors such as nuclease and lipases, which could be utilized as a switch to trigger stimuli‐responsive hydrogels.^[^ ^214^ ^]^ Wang and colleagues synthesized a DNA‐based hydrogel wound dressing to fight against the infection of *S. aureus*, thus reducing the unwanted bacteria‐induced inflammatory response. First, they prepared the cross‐linked nanostructure by DNA with specific sequence. Then, the polyanionic DNA nanostructure was combined with a cationic model antimicrobial L12 peptide which could restrain the proliferation and metabolism of *S. aureus* effectively. The L12‐loaded DNA hydrogel was rapid hydrolyzed when exposed to the endonucleases that secreted from *S. aureus* and the contents were released (Figure 12d).^[^ ^215^ ^]^

Likewise, Obuobi et al. proposed an anti‐inflammation drug delivery hydrogel system (vancomycin (Van)‐DNL), which contains a liposomal shell and loads DNA‐based nanogel (Van‐DNG) inside.^[^ ^216^ ^]^ The liposomal were degraded by the lipases that secreted by bacteria first, which lead to the slow release of Van‐DNG. Later, the DNA composed in Van‐DNG was hydrolyzed by DNase and consequently, the cross‐linked cationic antibiotic, Van, was released to the site where bacteria aggregated.

#### 4.4.4 Polysaccharide‐Based Enzyme‐Responsive Hydrogels

It has been proved that overactivated polysaccharase are closely related to some diseases and the massive decomposition of polysaccharide may lead to severe and irreversible consequences. In this background, there are numerous studies utilized the excessive polysaccharase as a switch to trigger the smart hydrogel for particular disease therapy.

De Jesus Perez and co‐workers realized effective treatment for idiopathic pulmonary fibrosis in mice and increased their survival rate successfully.^[^ ^217^ ^]^ Because of the abundant hyaluronidase and heparinase in the fibrotic environment, researchers developed an enzyme‐responsive hydrogel system (HH‐10) consisting of HA and heparin (HH) in response to the specific environment. In clinic, the key to the treatment of lung fibrosis is how to restrain inflammatory response effectively and how to prevent the fibrosis process. Hence, they selected IL‐10 as the drug to be delivered in which it could realize the anti‐inflammation and antifibrosis simultaneously. In the animal experiment, compared with IL‐10 or HH alone treated groups, the symptoms of pulmonary fibrosis were effectively relieved in the HH‐10 treated group after a 7‐days intratracheal administration. These results indicated that the HH‐10 hydrogel could be a promising candidate drug carrier to treat lung fibrosis alone or for the combination therapy.

## 5 External Factors Responsive Hydrogels for Inflammation Therapy

The endogenous factors responsive hydrogels are convenient, effective and have great potential for real clinical applications in which attract a lot research interest. Besides, aiming at the better controllability, the external factors responsive hydrogels are also exploited for inflammation therapy. In this section, we summarized several types of external factors responsive hydrogels with representativeness.

### 5.1 Thermosensitive Hydrogels

**Figure 13:**
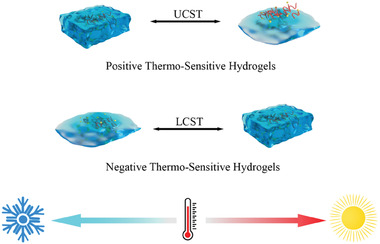
Classification of thermosensitive hydrogels and their phase change behaviors under different temperature.

Founded on the temperature difference between the human body and external environment or among different tissues and organs, the thermosensitive hydrogels are developed and has been one of the most studied stimuli‐responsive hydrogels. The sol–gel transition will occur to these hydrogels as the temperature changing. Materials used for the preparation of thermosensitive hydrogels generally contain both hydrophobic and hydrophilic components. And the phase transition behaviors of these hydrogels are depended on the rebalance of hydrophobic groups and hydrophilic groups.^[^ ^218^ ^]^ According to the different phase transition behavior as heating or cooling, thermosensitive hydrogels could be classified into positive thermosensitive hydrogels and negative thermosensitive hydrogels. The former will swell (sol state) at higher temperature (usually the human body temperature) but contract (gel state) at lower temperature (usually the room temperature) while the negative thermosensitive hydrogels are opposite (**Figure** **13**).

**Figure 14:**
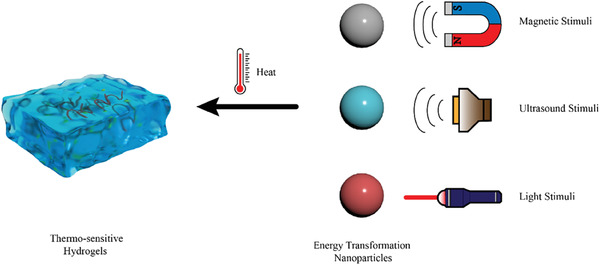
Various nanocomposite stimuli‐responsive hydrogels based on thermosensitive hydrogels and energy transformation nanoparticles.

Moreover, there also many studies combined thermosensitive hydrogels with other agents to form various stimuli‐responsive hydrogel systems. These incorporated agents could serve as a transducer that converting diverse forms of energy into heat. For example, Au NPs and carbon nanotubes convert light, into heat to turn on the transformation of thermosensitive hydrogels. And Fe_3_O_4_ NPs could transform the energy of magnetic force to thermal energy, thus trigger the action of hydrogels (**Figure** **14**).

#### 5.1.1 Positive Thermosensitive Hydrogels

The upper critical solution temperature is the most important parameter of positive thermosensitive hydrogels, which indicate their temperature threshold of gel‐to‐sol transition. The most frequently‐used materials to form this type of hydrogels are gelatin and some cellulose derivatives. In accordance with various demands, positive thermosensitive hydrogels could be applied as injection or wound dressing to realize inflammation suppression.

**Figure 15:**
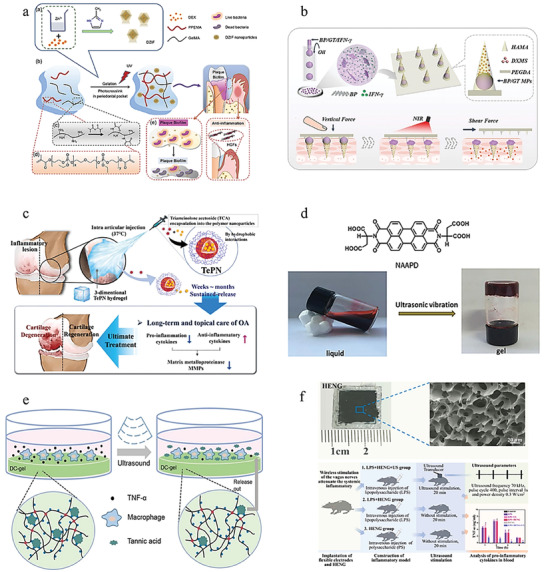
a–c) Thermosensitive hydrogels and d–f) ultrasound‐responsive hydrogels. (a) Zn^2+^ composed positive thermoresponsive hydrogel to achieve anti‐bacteria and periodontitis therapy. Reproduced with permission.^[^ ^127^ ^a]^ Copyright 2022, Elsevier. b) An NIR‐triggered hydrogel patch based on photothermal conversion to achieve dual drug delivery for SLE therapy. Reproduced with permission.^[^ ^220^ ^]^ Copyright, 2021 John Wiley & Sons. c) An injectable negative thermosensitive hydrogel for OA treatment. Reproduced with permission.^[^ ^221^ ^]^ Copyright 2021, Elsevier. d) NAAPD‐based ultrasound‐induced hydrogel. Reproduced with permission.^[^ ^222^ ^]^ Copyright 2021, Frontiers Media S.A. e) Ultrasound‐triggered hydrogel for tannic acid release to regulate macrophages. Reproduced with permission.^[^ ^223^ ^]^ Copyright 2022, Springer Nature Switzerland AG. f) A novel implantable ultrasound‐triggered battery‐free hydrogel nanogenerator to stimulate the vagus nerves to treat sepsis. Reproduced with permission.^[^ ^224^ ^]^ Copyright 2021, Elsevier.

For example, Luo and co‐workers created a nanocomposite hydrogel system (GelMA‐Z) for bacteria plaque‐induced periodontitis treatment. They start by preparing zeolitic imidazolate frameworks‐8 (ZIF‐8) NPs, and then loaded them in the thermosensitive hydrogel formed by GelMA. Thereafter, the injected pregel solution in the periodontal pockets transformed into hydrogel state under the irradiation of UV. And the network degraded gradually at 37 °C and the NPs were released to exert a therapeutic effect.^[^ ^219^ ^]^ Similarly, Jiang and co‐workers prepared a more advanced hydrogel system with almost the same ingredients. The DEX‐loaded (ZIF‐8) NPs were prepared and then doped in a hydrogel matrix (PGel), which consists of GelMA and methacrylic polyphosphoester (PPEMA), which contributes to the better thermostability, suitable swelling ratio, and mechanical property of this hydrogel system. Besides, the mesoporous ZIF‐8 NPs not only provide the drug loading space but also release Zn^2+^ continuously in the acidic inflammatory environment. The subsequent procedures were nearly consistent with the above: the hydrogel precursor solution was filled into the periodontal pocket and then exposed to the irradiation of UV to form hydrogels. Later, the NPs‐loaded PGel degraded slowly at oral temperature and the contents were released. Thanks to combined action of Zn^2+^ and DEX, this hydrogel exhibited a satisfactory antibacteria effect, thus the inflammatory symptoms were alleviated (**Figure** **15**a).^[^ ^127^ ^]^

Dong's group proposed a thermoresponsive hydrogel (Gel/PP–TA–Ag) wound dressing for anti‐inflammation, hemostatic, and against bacterial infection.^[^ ^225^ ^]^ This novel hydrogel mainly contains four parts: GelMA hydrogel matrix; tannic acid (TA) and polyphosphate (PP), both of which have the ability to promote coagulation; gallic acid modified Ag NPs, in which the gallic acid plays a crucial role in anti‐microbial while the Ag NPs contribute to photothermal conversion and the free Ag^+^ can also act as antibacterial agents. When under the exposure of near‐infrared (NIR) ray, the presence of Ag NPs leads to the temperature rise and accelerates the degradation of the hydrogel networks. Hence, more TA and Ag^+^ were released and the *S. aureus* were well cleared. The bacteria‐induced undesired inflammatory responses were restrained successfully.

Similarly, Zhao's group and Chuang's groups also developed thermoresponsive hydrogel therapy platform based on NIR and photothermal effect, respectively.^[^ ^220^ ^]^ Zhao and co‐workers proposed a separable and microspheres (MPs)‐composed microneedle (MN) patch for SLE treatment. The MNs were synthesized by methacrylated hyaluronic acid (HAMA), which has good biocompatibility. Meanwhile, the MPs were prepared with BP that contributed to photothermal conversion and gelatin, which is a commonly used thermoresponsive hydrogel material. As the patch exposed to NIR radiation, the heat generated by BP causes the gel–sol transition of the microspheres, thus weakening the connection between MNs and the patch. Thus, the MNs detach from the patch under the action of shear force and remain in the skin tissue for sustained drug (DEX) release. Furthermore, the MPs could also work as a thermoresponsive hydrogel drug vehicle to achieve the dual drug (Interferon γ) delivery simultaneously (Figure 15b). Chuang and co‐workers reported an injectable methylcellulose‐based hydrogel for RA therapy.^[^ ^226^ ^]^ The polypyrrole–polyethylenimine‐based NPs (PEI–PPY NPs) were prepared and served as photothermal converters. The temperature rise induced by the PEI–PPY NPs loosened the hydrogel network and the encapsulated strontium ranelate (SrR) and sodium chloride were released. Besides, the excessive ROS in the inflamed joint were metabolized more quickly due to the accelerated flow of blood induced by SrR. The combination of the photothermal and drug therapy based on this hydrogel system leads to the effective alleviation of arthritis.

Wu and co‐workers proposed a hydrogel system for asthma management.^[^ ^227^ ^]^ This hydrogels system was prepared by P407, Carbopol 974P NF, and Polyoxyl 15 hydroxystearate, and could transform from sol state into gel under 33 °C, which could be applied as a vaccine carrier through nasal delivery. By loading allergens and FK506, which is a commonly used immunosuppressant for asthma, inside, this system could induce immune tolerance effectively.

#### 5.1.2 Negative Thermosensitive Hydrogels

As opposed to what introduced above, the most crucial indicator of negative thermosensitive hydrogels is the LCST. While the LCST describes the temperature threshold of sol‐to‐gel transition of negative thermosensitive hydrogels. Compared with the positive thermosensitive hydrogels, there are more diversified materials for the preparation of negative thermosensitive hydrogels, such as previously mentioned pNiPAAm, PVA, PEG/PLGA copolymers, and cellulose. This type of hydrogels is usually developed as injection or oral drug carriers to achieve sustained drug release and may have some specific functions for particular inflammation treatment.

The negative thermosensitive hydrogels are irreplaceable in the development of a novel therapy platform for the treatment of arthritis. The work principle and design idea of them are almost consistent: first, prepared a hydrogel precursor which is liquid at room temperature; then, the pregel will transform to hydrogel at ≈37 °C and realize sustained drug delivery; besides, these hydrogels usually possess mechanical properties that are similar to synovial fluid. For example, Song and co‐workers synthesized an injectable polymeric nanoparticle (TePNs) for long‐term management of OA.^[^ ^221^ ^]^ The TePNs were prepared by an amphiphilic PNP and loaded an NSAID, triamcinolone acetonide (TA), inside. Furthermore, the TePNs could present sol–gel transition at the human body temperature since intra‐articular injection. The hydrogel formed by TePNs filled the diseased joint to achieve sustained drug administration and due to its lubrication and mechanical property, this hydrogel could protect the joint from physical friction, thereby reducing further cartilage degradation (Figure 15c). Additionally, Lanceros‐Mendez and co‐workers proposed a hydrogel system by using genipin to combine CS and β‐glycerol phosphate disodium salt for controlled release of DS to alleviate the inflammatory response of OA.^[^ ^228^ ^]^ Zang and co‐workers investigated a P407, HA, and chondroitin sulfate E disaccharide (ΔUA‐diSE) composed injectable hydrogel for better treatment of OA.^[^ ^87^ ^]^ While the ΔUA‐diSE could worked as mimic synovial fluid and has been verified helpful to regulate the expression of C5b‐9, which is an inflammation‐related factor about OA.

For RA therapy, Lee and colleagues reported an injectable nanocomposite hydrogel (CMM) to realize chemo‐photothermal combination therapy for RA. The CS, gelatin, and β‐glycerophosphate constitute the hydrogel matrix while the melanin plays a part in photothermal conversion and MTX acts as a chemical therapy agent. The CMM in sol state was injected into RA‐induced joint and maintain its state at interarticular temperature. Following by the irradiation of NIR laser (808 nm, 0.5 W cm^−2^, 3 min), the CMM transformed to hydrogel by sol–gel transition. The temperature rising not only is beneficial for pain relief and bone regeneration but also induces MTX releasing which controls the RA symptom further.^[^ ^229^ ^]^ Gao and colleagues developed a bio‐photothermal combination therapeutic hydrogel by combining the black phosphorus nanosheets (BPNs) into thermosensitive platelet‐rich plasma (PRP)–CS hydrogels.^[^ ^230^ ^]^ Likewise, when under the exposure of NIR laser, the fluent BPN/CS/PRP transformed into stable hydrogel due to the heat produced by BPNs. As the gradual degradation of the hydrogel networks, more BPNs and PRP were released and promoted the osteogenesis, as a result the RA was alleviated to a certain extent.

In addition to arthritis treatment, the negative thermosensitive hydrogels are also exploited for local drug administration. In order to treat oral lichen planus, Wojcicki and co‐workers proposed a P407‐based hydrogel to accomplish the long‐term administration of a hydrophobic anti‐inflammatory drug, dexamethasone acetate (DMA).^[^ ^231^ ^]^ The DMA was incorporated into the P407‐hydroxypropyl‐β‐cyclodextrin (HP‐β‐CD) cross‐linked hydrogel and obtained a suitable gelation temperature by adjusting the weight percentage of P407. This solution became hydrogel after intraoral dosing and worked as a sustained‐release mucoadhesive agent for inflammation alleviation. Little and co‐workers carried out a novel hydrogel system, TEMPS (which means thermogel, extended‐release microsphere‐based‐delivery to the paranasal sinuses), whose LCST is ≈35 °C, for sustained mometasone furoate release.^[^ ^232^ ^]^ The matrix of TEMPS was made up by pNiPAAm which is a frequently used temperature‐responsive biomaterial. Since the formation of TEMPS hydrogels, the drug released time was delayed to 4 weeks. Compared with conventional nasal steroid sprays, drugs administered by TEMPS are less frequency and have higher utilization ratio of drug molecules. Aiming to improve the bioavailability of Kangfuxin liquid (KFX), Xu's group proposed a P407‐KFX mixed hydrogel system.^[^ ^233^ ^]^ The P407 could completely dissolve into KFX at 4 °C and present gelation under 37 °C. Thanks to the encapsulation and protection of the hydrogel networks, both the drug availability and therapeutic effect for UC were significantly increased after oral delivery.

Sung and co‐workers developed a bioinspired hydrogel spray (Pe‐hydrogel) for all‐in‐one treatment of inflammatory bowel disease (IBD), which could be administrated at the same time as endoscopy.^[^ ^201^ ^]^ This hydrogel has two main parts: one is the negative thermoresponsive hydrogel matrix formed by methoxy poly(ethylene glycol) (mPEG) and PCL; another one is a peptide with a particular sequence. Then the synthesized peptide was conjugated onto the hydrogel to get the Pe‐hydrogel. This peptide was designed to mimic the D1 domain on the flagellin of Bacillus subtilis, which acts as the primary binding site for the Toll‐like receptor 5 (TLR‐5). Besides, the binding of flagellin and TLR‐5 usually leads to intestine inflammation and mucosa damage. Hence, the combination between the Pe‐hydrogel and TLR‐5 could significantly relieve the inflammatory response, which is the principle of the drug‐free therapy induced by Pe‐hydrogel. The adhesive gelation could also work as a barrier to counter the enhanced permeability in diseased intestine. In the next experiments, the hydrogel was able to enhance the transmembrane capacity of anti‐inflammatory drug molecules (DEX), which improved the therapeutic effect further. Through the tests based on human colorectal cell line, clinical IBD patient cells, gut‐on‐a‐chip, mouse IBD models, and pig, they further demonstrated the great potential of the Pe‐hydrogel for clinical translation. Huang and co‐workers developed an injectable HA‐based supramolecular hybrid hydrogel to delivery IL‐1β stimulated exosomes, which could further target the inflammatory part in brain. The sol–gel transformation would occur when temperature exceeds 26 °C. Through the formation of hydrogel, the retention time of exosomes could be prolonged and thus help neuroinflammation inhibition and neuronal recovery.^[^ ^234^ ^]^

### 5.2 Ultrasound‐Responsive Hydrogels

By reason of the noninvasiveness, good controllability, safety as well as the relatively mature technique, ultrasound has been widely applied in clinics such as ultrasonic imaging and focused ultrasound surgery. Currently, combining ultrasonic stimuli with smart hydrogel system, ultrasound‐triggered hydrogels are also researched a lot for cancer therapy, clinical diagnosis, ultrasonic imaging, biosensors, and so on.^[^ ^235^ ^]^ According to the different role of ultrasound, the ultrasound‐responsive hydrogels could be further divided into ultrasound‐induced hydrogels and ultrasound‐triggered hydrogels. The phase change of them primarily depends on the destruction or formation of bonds or physical cross‐linking in the hydrogel networks that induced by ultrasonic waves. As for inflammation treatment, ultrasound‐responsive hydrogels are mostly explored as drug delivery platforms to accomplish on‐demand drug release.

#### 5.2.1 Ultrasound‐Induced Hydrogels

Because of the unsatisfactory property like stress and toughness of the hydrogels which synthesized by conventional methods, scientists developed the ultrasound‐induced hydrogels to obtain improved performance. The formation of the interaction, such as hydrogen bonds, between molecules will lead the hydrogel precursor to transform into a gel state when stimulated by ultrasonic waves.

The schistosoma japonicum is a harmful parasite in the human body with high infectivity, which could induce a variety of chronic or acute inflammations and even cause death in severe cases. Nowadays, there are still a lot of people who are exposed to the risk of schistosome infection. On these grounds, Cheng and co‐workers reported an ultrasound‐induced amino‐acid‐based hydrogel for long‐term drug release. The amphiphilic precursor, *N*,*N*′‐diaspartic acid 3,4,9,10‐tetracarboxylic diimide (NAAPD), formed hydrogel exhibited great strength due to the formation of hydrogen bond and π–π interaction. The Niclosamide derivative, which is an effective anticercarial drug, was loaded in to the NAAPD formed hydrogel networks. Later, in vitro test, this hydrogel released drug molecules slowly and prolonged the drug‐released period to about 15 days. Which demonstrates the favorable prospect of this hydrogel for the restrain of schistosomes (Figure 15d).^[^ ^222^ ^]^

#### 5.2.2 Ultrasound‐Triggered Hydrogels

The ultrasound‐triggered hydrogels are contrary to the above, which could undergo the phase change, or realize specific functions by interacting with other components in response to the excitation of ultrasonic wave. Besides drug delivery systems, there also many researchers utilized these hydrogels to design nanogenerators to achieve the electrical stimulation of the vagus nerves, which is an emerging clinical treatment for systematic inflammation or autoimmune disease with great potential.

For instance, Cao and co‐workers reported a dual cross‐linked hydrogel (DC‐gels), which could be a candidate drug delivery system for the remedy of various inflammations on load‐bearing tissues such as muscle and cartilage.^[^ ^223^ ^]^ The backbone was obtained by mixing HA with four‐armed polyethylene‐glycol acrylate (4arm‐PEG‐Aclt). To loading TA inside, the 4‐(aminomethyl) phynyl boronic acid was introduced to provide the binding sites. The dynamic covalent bonds which formed between TA and DC‐gel could respond to the mechanical stimuli of ultrasound. In their vitro experiments, the release of TA molecules triggered by ultrasound suppress the macrophage induced inflammatory response effectively. Besides, they confirmed that the DC‐gels would not respond to the mechanical stimuli under normal conditions, by which to avoid the undesired drug leakage (Figure 15e). Hayes and co‐workers developed a controllable cross‐linking CS hydrogel with reversible Diels–Alder linkers for on‐demand protein delivery.^[^ ^236^ ^]^ When stimulated by focused ultrasound, this hydrogel will undergo a retro Diels–Alder reaction, which leads to the protein release. By loading anti‐inflammatory protein or peptide drugs inside, this drug delivery platform may be applied for inflammation therapy.

Luo and co‐workers presented an implantable, programmable, and battery‐free hydrogel nanogenerator (HENG), in which PAAM hydrogel matrix served as liquid phase while the embedded graphene served as collection electrode. Excited by external ultrasound induced compressive force, the HENG is able to generate enough alternating electric current to stimulate the vagus nerves. And then, the electrical signals were transmitted to the spleen, further affecting the immune organs as well as immune cells.^[^ ^237^ ^]^ By adjusting the parameter of ultrasonic pulse such as frequency and time, the generated electric current could be controlled.^[^ ^224^ ^]^ They implanted this hydrogel nanogenerator into animal models and as anticipated, the remarkable suppression of sepsis was observed later, which indicated the possibility of HENG for systemic inflammation management (Figure 15f).

### 5.3 Magnetic‐Responsive Hydrogels

The magnetic‐responsive hydrogel nanocomposites are the combination of polymer hydrogel matrix and magnetic NPs, which are represented by Fe_3_O_4_. Ordinarily, in order to incorporate the magnetic NPs into these hydrogels, three methods are frequently‐used: i) blending method, ii) in situ precipitation method, and iii) grafting‐onto method.^[^ ^238^ ^]^ Owing to the superb precise targeted capacity and controllability, magnetic‐responsive has been developed as soft robots, smart drug delivery systems, and tissue engineering scaffolds.^[^ ^239^ ^]^

**Figure 16:**
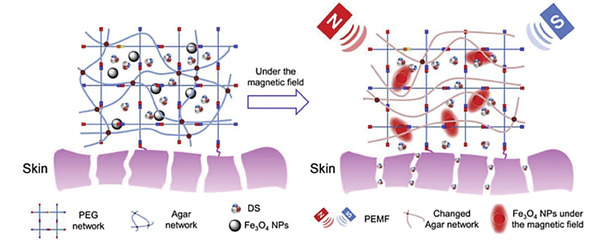
Magnetic‐responsive hydrogels. Fe_3_O_4_ nanoparticles embedded hydrogel wound dressing to promote soft tissue repairing. Reproduced with permission.^[^ ^241^ ^]^ Copyright 2022, Elsevier.

In 2014, Tombácz and co‐workers reported an injectable magnetic‐responsive hydrogel as a potential material for osteoarthritis treatment.^[^ ^240^ ^]^ In their study, the chondroitin sulfate A‐coated magnetic NPs were loaded into the HA hydrogel matrix by in situ precipitation method. This hydrogel could respond to the implanted permanent magnet and achieved drug release. Yang and co‐workers incorporated Fe_3_O_4_ NPs into a tetra‐PEG/agar (PA) hydrogel matrix to obtain a magnetic‐responsive hydrogel dressing (PA/Fe_3_O_4_/DS).^[^ ^241^ ^]^ Loading the DS into the hydrogel networks, this system could be used for the treatment of soft tissue injury induced inflammation. When the PA/Fe_3_O_4_/DS hydrogel was placed in a pulsed electromagnetic field, the DS molecules were released by two routes, which induced by Fe_3_O_4_ NPs. One is the shake triggered by magnetic field and the other one is that the heat generated by Fe_3_O_4_ accelerates the melt of agar, both of which lead to the collapse of the hydrogel networks. The animal experiments further demonstrated that the PA/Fe_3_O_4_/DS not only suppressed the inflammatory response effectively but also promoted tissue repair under the magnetic stimuli (**Figure** **16**). Zhang and co‐workers proposed a novel hydrogel wound dressing for the promotion of tendon injury healing.^[^ ^242^ ^]^ They prepared a Fe_3_O_4_ encapsulated gelatin hydrogel to load the celecoxib. And same as the above, the electromagnetic field boosts the degradation of the hydrogel and the released celecoxib further acts on the macrophages. As the increase of M2 macrophages, remarkable inflammation alleviation and wound healing were observed in the model animals.

Cheng and co‐workers prepared an implantable magnetic‐responsive hydrogel by grafting amino‐terminated superparamagnetic magnetic NPs onto collagen fibers under the action of genipin.^[^ ^243^ ^]^ Being different from conventional anti‐inflammation platforms, this hydrogel could induce directional polarization of macrophages to M2 phenotype by the magnetism‐induced nano/microforces, thereby rebalance the M1/M2 concentration to an optimal state through the podosome/Rho/ROCK mechanical pathway. As mentioned previously, M2 macrophages could produce anti‐inflammatory cytokines like TGF‐β and IL‐6 to restrain the inflammation and promote the tissue regeneration. Besides, by adjusting the magnetic field, remotely time‐scheduled macrophage polarization came true.

Furthermore, as an emerging drug delivery platform, magnetic‐responsive hydrogel‐based soft robotics has gained more and more attraction due to its excellent flexibility and controllability. They are mainly produced by 3D/4D printing. And thanks to the magnetism anisotropy, they are capable of responding to the magnetic stimulation sensitively, which could realize target drug delivery precisely.^[^ ^244^, ^245^ ^]^ However, the magnetic‐responsive soft robots are mostly applied for the therapy of cancers. For inflammation management, more research is needed.

### 5.4 Light‐Responsive Hydrogels

Light‐responsive hydrogels could under a phase change in respond to light with specific wavelength (NIR, UV or visible light) due to the presence of photosensitive groups. Through light irradiation, the remote, noncontact and precise control for light‐responsive hydrogels could be realized. Therefore, light‐responsive hydrogels have been applied for a lot of fields such as stereo lithography appearance 3D printing and drug carriers.^[^ ^246^ ^]^ Similar to the ultrasound‐responsive hydrogels, based on the phase change behavior, light‐responsive hydrogels could be classified into light‐induced hydrogels and light‐triggered hydrogels. Additionally, as we will introduce later, the light‐responsive hydrogels are mostly applied for inflammation control of skin or other superficial tissues. These are mainly ascribed to the weak penetration of light, which is also a limitation that still needs more effort to overcome.

#### 5.4.1 Light‐Induced Hydrogels

The common characteristic of light‐induced hydrogels is that when exposed to light, the hydrogel solution could transform into solid or gel state mainly due to the dimerization or isomerization triggered by photosensitive groups. And thereby, they are suitable to form the shape‐match hydrogels based on different demands.

In the study reported by Xia and co‐workers, an HA‐based hydrogel was developed to reduce the level of proinflammatory cytokine and promote wound healing, which could be a promising candidate for diabetic wound management.^[^ ^247^ ^]^ The HA hydrogel matrix was modified by methacrylic anhydride and N‐(2‐aminoethyl)‐4‐[4‐(hydroxymethyl)‐2‐methoxy‐5‐nitrophenoxy]‐butana­mide (NB) groups to obtain HA–MA–NB hydrogels (HNM). Later, the lyophilized amnion‐derived conditioned medium (AM‐CM), which could significantly accelerate the wound repairing process due to the presence of abundant active proteins and other biomolecules, was encapsulated in the HNM hydrogels. Then, the HNM‐M hydrogel precursor was injected into the wound and photopolymerize within 3 s under the irradiation of UV. In the animal models, this hydrogel wound dressing adheres tightly to the skin and hastens the diabetic wound healing of mice effectively.

**Figure 17:**
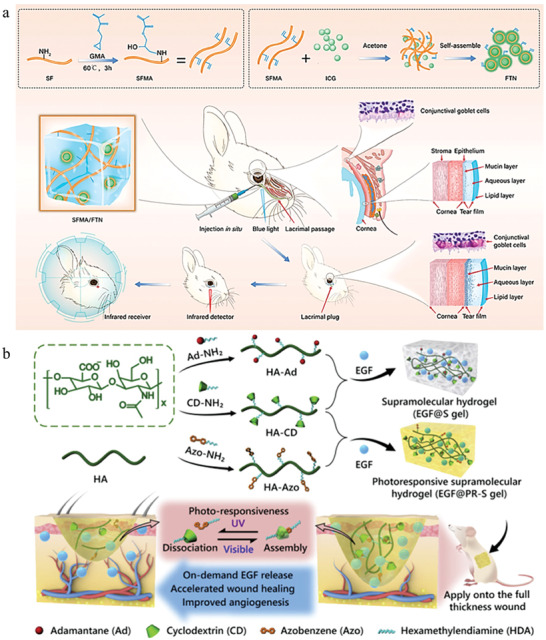
Light‐responsive hydrogels. a) Visible‐induced hydrogel to alleviate dry eye and could be monitored by NIR due to the presence of ICG. Reproduced with permission.^[^ ^248^ ^]^ Copyright 2022, John Wiley & Sons. b) UV‐triggered HA‐based hydrogel to accelerate wound healing. Reproduced with permission.^[^ ^249^ ^]^ Copyright 2020, Elsevier.

Chen and co‐workers developed a in situ forming hydrogel to serve as a novel absorbable lacrimal plug for the alleviation of dry eye, which is a common chronic disease with complex pathogenesis.^[^ ^248^ ^]^ They used methacrylate‐modified silk fibroin (SFMA) as the hydrogel matrix and, to achieve the noninvasive monitor, NIR‐triggered indocyanine green (ICG) was prepared as fluorescence tracer NPs (FTN). The SFMA/FTN hydrogel solution was injected into the canaliculi, exposed to the blue light, the liquid quickly transformed into hydrogel and formed a shape‐match plug to block the lacrimal passage. Therefore, the retention time of tears on the eye surface was prolonged. Moreover, the morphology and therapeutic effect of SFMA/FTN hydrogels could be monitored in real time by ICG‐induced NIR imaging (**Figure** **17**a).

#### 5.4.2 Light‐Triggered Hydrogels

The cleavage reaction or isomerization that happened to the light‐triggered hydrogels will lead to the collapse of hydrogel networks and thus, stimuli‐responsive drug release is realized. Several kinds of photosensitive agents, such as diazobenzene (Azo) and spiropyrane, could change the hydrogel network from hydrophobicity to hydrophilicity, which further leads to the swelling of hydrogels. Besides, a lot of agents possess light‐cleavable bonds, which could also lead to the hydrogel degradation.

Xu and colleagues investigated an HA‐based photoresponsive supramolecular hydrogel (EGF@PR‐S gel) wound dressing to remove unfavorable inflammatory response and promote wound healing.^[^ ^249^ ^]^ In this research, they grafted Azo and β‐CD onto the chain of HA, respectively. Under visible light, the Azo has the capacity to cross‐link with the hydrophobic cavity of β‐CD, which resulted in the formation of a host–guest linkage. When exposed to the UV, the structure change of Azo destroyed the linkage, which further made the hydrogel network loosened and the loaded epidermal growth factor (EGF) was released to accelerate the wound healing (Figure 17b).

Including the above, conventional wound dressing is lake of the ability to monitor bacterial infection or wound recovery degree. Inspired by these, smart wound dressings were invented. Ma and co‐workers developed an NIR‐triggered hydrogel for infection monitoring and bacteria elimination.^[^ ^250^ ^]^ The matrix was made up by PEG and PVA while a common antibiotic, gentamicin sulfate, was grafted onto the PEG chains through a UV‐cleavable linker called 1‐(5‐methoxy‐2‐nitro‐4‐prop‐2‐ynyloxyphenyl) ethyl *N*‐succinimidyl carbonate. Furthermore, Cy3 and Cy5 modified silica NPs (SNP‐Cy3/Cy5) were encapsulate into the hydrogel to serve as a fluorescence probe because of their fluorescence intensity change when in an acidic microenvironment induced by infected bacteria. A kind of upconversion NPs were also loaded to transform the NIR excitation (980 nm) into a UV (365 nm) to derive the action of this hydrogel system. This smart wound dressing presented a remarkable antibacterial effect and avoided excessive inflammation. Additionally, there are researchers further combined the UV‐triggered hydrogel with flexible electronics to dual‐layer smart wound dressing.^[^ ^251^ ^]^ Hence, smart hydrogel wound dressing with more precise monitoring and more convenient use were obtained, which provides an innovative strategy for the recovery of chronic wounds.

Zhao et al. reported a visible light‐triggered BMSCs‐loaded hydrogel (DCS‐RuB2A2) to promote oral wound healing.^[^ ^252^ ^]^ The CS and the Ru bipyridine which is a photo‐cross‐linking agent were used to prepare the hydrogel. In the dark oral, the hydrogel would cover the wound to inhibit the bacterial infection. After visible light irradiation, the picolinaldehyde group detached from the molecular chains, which lead to the collapse of the hydrogel and the BMSCs were then released to recruitment abundant red blood cells to the damaged tissue and contributed to the formation of blood clotting. Besides, the Ru bipyridine served as an antibacterial agent in this process. The DCS‐RuB2A2 hydrogel created a humid, sterile, and temperature‐appropriate environment on the wound of oral mucosal, which greatly benefit to the wound recovery and inflammation suppression.

## 6 Multifactor‐Responsive Hydrogels for Inflammation Therapy

In the past few years, a lot of researchers dedicated their efforts to combine different stimuli factors to develop various multifactor‐responsive hydrogels. Due to the type of combined stimuli factors, we classified the multifactor‐responsive hydrogels into two categories: i) multiendogenous factors‐responsive hydrogels and ii) endogenous‐external factors‐responsive hydrogels. Most of the multiendogenous factors‐responsive hydrogels aimed at enhanced response sensitivity, response speed or targeting capacity. And endogenous‐external factors‐responsive hydrogels could incorporate both the convenience of endogenous factors‐responsive hydrogels and better controllability of external factors‐responsive hydrogels. These multifactor‐responsive hydrogels provide a new insight for effective and precise treatment of inflammations. In this section, we summarized recent studies about multifactor‐responsive hydrogel system for inflammation therapy.

### 6.1 Multiendogenous Factors‐Responsive Hydrogels

Pandey et al. prepared an enzyme/pH‐responsive hydrogel by cross‐linking pectin and PAAm for oral delivery of BUD for UC therapy.^[^ ^253^ ^]^ BUD has short retention time in the human body, which usually leads to unsatisfactory therapeutic effects. As mentioned previously, the PAAm could endow this hydrogel system with pH‐sensitivity due to the amide groups (─CONH_2_) on its molecular chains. While the pectin could be degraded only with the catalyzation of some enzymes in the colon. Thereby, this hydrogel was contracted in the stomach to avoid drug leakage but loosed in the colonic environment because of the combined action of pH and enzyme.

**Figure 18:**
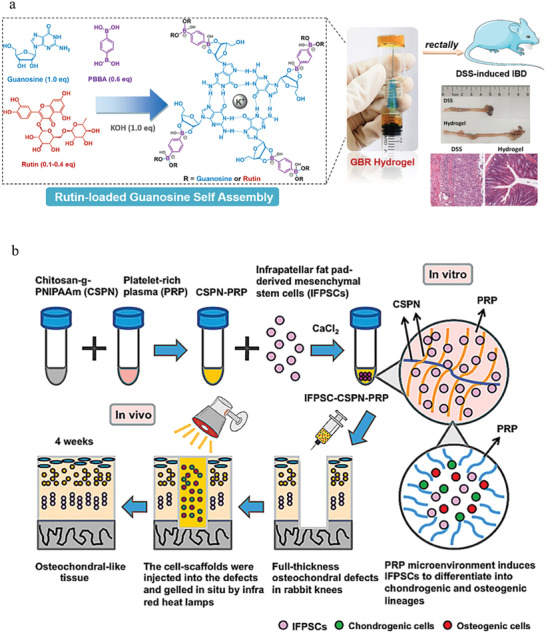
Multiendogenous factors‐responsive hydrogels. a) Injectable pH/ROS‐responsive hydrogel for IBD therapy. Reproduced with permission.^[^ ^254^ ^]^ Copyright 2022, American Chemical Society. b) An enzyme/thermoresponsive hydrogel to induce mesenchymal stem cell differentiation for osteochondral lesions caused by arthritis. Reproduced with permission.^[^ ^255^ ^]^ Copyright 2022, Elsevier.

Li and co‐workers reported a rectal injectable hydrogel (GBR) to improve the low bioavailability of rutin, which could alleviate the IBD effectively.^[^ ^254^ ^]^ The rutin was cross‐linked with guanosine by a borate ester linkage, which could response to both acidic and ROS‐excessive environment. In their following experiments, this GBR hydrogel released drug molecules rapidly and precisely to the inflamed tissues and significantly alleviated the DSS‐induced colitis in mice, which indicated the possibility of this hydrogel drug carrier to delivery anti‐inflammatory drugs with poor aqueous solubility and stability for IBD treatment (**Figure** **18**a).

Yeh and colleagues developed a thermoresponsive enzyme‐degradable hydrogel scaffold (CSPN‐PRP) to induce mesenchymal stem cell differentiation for osteochondral lesions caused by arthritis.^[^ ^255^ ^]^ The CS‐graft‐pNiPAAm (CSPN) was used as the thermosensitive hydrogel matrix to load infrapatellar fat pad‐derived mesenchymal stem cell (IFPSC) and PRP. The hydrogel solution was injected into the damaged joint and undergoes a sol–gel transition to form a stable hydrogel scaffold under the action of body temperature. Moreover, the PRP contains various cytokines and could provide a suitable environment for IFPSC growth and differentiation, which could promote the chondrogenesis in OA chondrocytes. In the design of tissue scaffolds, the rate of scaffold degradation should match which of tissue regeneration. Hence, in this work, the incorporation of amide linkage in the hydrogel network allowed the hydrolyzation induced by lysozyme, which leads to a balanced rate of degradation and resorption. The micromolecular fragments of CSPN‐PRP hydrogel, which generated after the enzymatic decomposition were expelled by renal which indicate the low‐toxicity of this hydrogel. In the later animal experiments, using rabbits as models, researchers found that the CSPN hydrogel scaffold facilitated the osteogenesis effectively (Figure 18b).

Cao and co‐workers investigated an ROS/pH‐responsive hydrogel depot (EGCG HYPOT) to restrain oxidative stress and control inflammation.^[^ ^256^ ^]^ Epigallocatechin‐3‐gallate (EGCG), which characterized by good antioxidant and anti‐inflammatory capacity, was used to synthesize a phenylborate ester bond‐contained copolymer (HAMA‐PBA) with PBA‐modified methacrylated HA (HAMA). And the phenylboronic acid–diol complex could respond to both ROS and acidic environment of inflammations, thus realize a local and sustained release of EGCG molecule. Under the irradiation of UV (≈365 nm, 10 s), the HAMA‐PBA was cross‐linked with GelMA and obtained enhanced mechanical properties and adhesivity. Accompanied with the shape adaptability, the EGCG HYPOT may be used to treat OA, IVDD or periodontitis.

### 6.2 Endogenous‐External Factors‐Responsive Hydrogels

**Figure 19:**
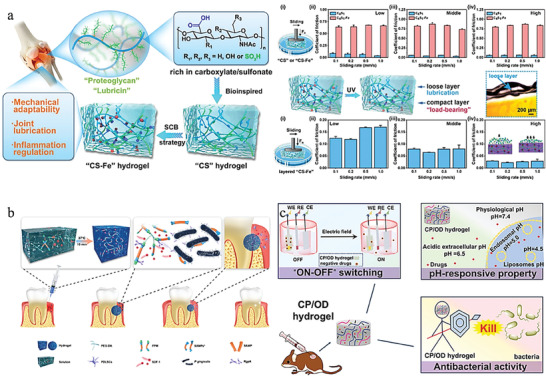
Endogenous‐external factors‐responsive hydrogels. a) A layered UV‐triggered ROS/NO‐responsive hydrogel for OA treatment. Reproduced with permission.^[^ ^257^ ^]^ Copyright 2022, American Chemical Society. b) An enzyme/thermoresponsive hydrogel for periodontitis therapy by restrain the pathogenic bacteria. Reproduced with permission.^[^ ^259^ ^]^ Copyright 2021, American Chemical Society. c) An injectable CS‐based electric/pH‐responsive hydrogel for drug local delivery. Reproduced with permission.^[^ ^262^ ^]^ Copyright 2022, Elsevier.

Xie and co‐workers developed a layered UV‐triggered ROS/NO‐responsive hydrogel (“CS”‐Fe) with similar mechanical property to normal cartilage for the management of OA‐induced cartilage degeneration.^[^ ^257^ ^]^ This hydrogel primarily contains carboxylates/sulfonates formed hydrogel matrix, which is tightly cross‐linked by abundant Fe^3+^. The carboxylates and sulfonates are rich presence in healthy cartilage while Fe^3+^ endows this hydrogel better toughness and load‐bearing capacity. Besides, the interconversion between Fe^3+^ and Fe^2+^ could scavenge overproduced ROS and NO in diseased joint. To get the layered “CS”‐Fe hydrogel, the prepared “CS”‐Fe hydrogel was put under the irradiation of UV, which could transform Fe^3+^ to Fe^2+^. In this way, a lubricating superficial zone and a load‐bearing deep zone were created. In the mechanical property test, the “CS”‐Fe hydrogel well met the mechanical requirements of human joints in both walking and running. Furthermore, the layered “CS”‐Fe hydrogel also avoided cell death of chondrocytes in sliding tests. Hitherto, a cartilage‐inspired hydrogel with mechanical adaptability, joint lubrication, and inflammation regulation capacity was investigated successfully by researchers (**Figure** **19**a).

Fan and colleagues reported a drug encapsulated thermo/enzyme‐responsive hydrogel to alleviate OA through autophagy regulation.^[^ ^258^ ^]^ First, Sinomenium (SIN), which is an extract from a Chinese medical plant called Sinomenium acutum and used to treat patients with OA or RA in clinic, was loaded in CS‐formed microsphere whose size decided the release rate of drug molecules. Then the drug‐loaded CS microspheres were encapsulated in GelMA hydrogel matrix by photoinduced cross‐linking. As we summarized previously, as a derivative of gelatin, GelMA could be degraded by collagenase and have quite thermosensitivity. Through the combined action of the human body temperature and excessive MMP‐13 (a kind of collagenase) in OA‐induced joint, the hydrogel swelled rapidly and got equilibrium in 36 h. In their later test, there are still 24.7% of drugs remained in the hydrogel network after 20 days, which indicated the ability of this novel GelMA‐based hydrogel to achieve sustained drug release. In OA mice models, the released SIN molecules remarkably repress the expression of MMP‐13 and ADAMTS‐5 (a protein which may lead to cartilage degeneration), which demonstrated the persistent and effective alleviation of OA.

Gingipain, including arginine protease (Rgp) and lysine gingipain, has been verified closely related to alveolar bone loss and bleeding tendency in periodontitis gingiva and are secreted by Porphyromonas gingivalis, which is the most pathogen of periodontitis. Inspired by this, Ge and co‐workers reported an injectable hydrogel (PEGPD@SDF‐1) to realize tissue regeneration and periodontitis treatment in response to oral temperature and gingipain.^[^ ^259^ ^]^ The PEGPD@SDF‐1 hydrogel contains three major parts: i) four‐arm PEGDA were served as the scaffold of hydrogel; ii) a functional peptide module was designed which has a short antimicrobial peptide (SAMP) center and two anchor peptide units in its lateral. The SAMP possesses excellent antibacterial activity while the anchor peptide provides an Rgp‐cleavable site to response to the overexpressed gingipain; and iii) stromal cell derived factor‐1 (SDF‐1), which could recruit endogenous periodontal ligament stem cells to the inflamed sites, thereby promoting the tissue regeneration simultaneously. The PEGPD@SDF‐1 hydrogel solution was injected into the periodontal pocket of rats, then because of the effect of oral temperature (≈37 °C) the liquid state hydrogel underwent a sol–gel transition within 10 min. In their later H&E staining analysis, considerable bone regeneration was observed in PEGPD@SDF‐1 treated group compared with other groups. And the proliferation and metabolism of P. gingivalis were also suppressed significantly (Figure 19b).

Mao and co‐workers reported a microporous pH/thermoresponsive hydrogel wound dressing (carboxymethyl agarose (CMA)‐Ag) to suppress bacteria‐induced inflammation and accelerate the wound healing procedure.^[^ ^260^ ^]^ The hydrogel matrix was prepared by CMA, whose molecular chains were cross‐linked by hydrogen bonding to form the network. A large number of carboxyl and hydroxyl groups provide combining sites for Ag^+^, which further improves the strength of the cross‐linked hydrogel, in which the hydroxyl group complex with Ag^+^ while the carboxyl group forms an organic–metal structure (─COOAg). Both the acidic environment and higher temperature could destroy the complexation between hydroxyl and Ag^+^. Besides, H^+^ could also lead to the degradation of ─COOAg, which generates abundant free Ag^+^. In the animal experiment, thanks to the prolonged stable release of Ag^+^, the CMA‐Ag hydrogel exhibited excellent antibacterial activity in the *S. aureus*‐infected wound and the damaged skin completely healed within 14 days, which was earlier than other groups. The researchers also indicated the good cytocompatibility and hemocompatibility of CAM‐Ag hydrogel.

Wang and colleagues reported a nanocomposite hydrogel system for the administration of MR409, which is a hormone analog peptide that could regulate the level of ROS and plays a role in the treatment of disk degeneration.^[^ ^261^ ^]^ This hydrogel system was composed of a thermosensitive hydrogel matrix, which is prepared with PLGA–PEG–PLGA and a self‐assembly MR409‐loaded ROS‐responsive vesicle, which is prepared with methoxy poly(ethylene glycol)‐*b*‐poly(propylene sulfide) (PPS–PEG). As the increase of environment temperature after injecting, the hydrogel network collapsed gradually and as a result the MR409‐loaded vesicle was released. The ROS in the diseased intervertebral disk transformed the hydrophobic part (PPS) of the vesicle into a hydrophilic part, thereby leading to the disintegration of the vesicle. The suppressed tripartite motif‐containing 16 (TRIM16) expression and activated aggrecan (ACAN) expression indicated the alleviation of disk degeneration in rats conjointly. This research proposed a new strategy to solve the issue that protein drugs are easy to be degraded during the drug administration and provided a novel approach for intervertebral disk degeneration therapy.

Guo and co‐workers developed an injectable electric/pH‐responsive hydrogel (CP/OD) for drug local delivery.^[^ ^262^ ^]^ CS and polyaniline, both of them possess a certain electrical conductivity, were used to prepare a CS‐*g*‐polyaniline CP. The CP polymers were mixed with OD to form the cross‐linked hydrogel network and due to the generation of Schiff base bonds between the ─NH2 and ─CHO that derived from CP and OD, this hydrogel could respond to acidic environment. Besides, the external electric field drove the migration of the charged molecules and accompanied with the polyaniline reduction‐induced change of the overall net charge in the hydrogel, the negatively charged IBF and amoxicillin molecules were released. And the drug cumulative release was related to the voltage, which was 69% at 1 V and 82% at 3 V. This CP/OD hydrogel showed both better controllability and capacity for sustained and precise local drug release, which could be an ideal candidate drug carrier for inflammation management (Figure 19c).

## 7 Conclusion and Outlook

With the rapid advance of material science and biotechnology, the design and application of smart hydrogel has been one of the most topic areas of biomedical engineering. In this review, we introduced and summarized various applications of smart hydrogel systems in the treatment of inflammations, including oral/injectable drug carriers, wound dressing, eye drops, tissue engineering scaffolds, spray, and so on. We believe that the greatest advantage of smart hydrogels for inflammation therapy is that they significantly improve some frequent shortcomings of anti‐inflammatory drugs, such as poor solubility, poor targeting capacity, short retention time, burst release, ease of decomposition in vivo, etc. While the development of a new drug usually requires a complex process that often takes several decades. However, not only does the development of smart hydrogels require a shorter period, but a type of stimuli‐responsive hydrogel could often be used to deliver multiple drugs. While improving the drug bioavailability, patient compliance, and treatment effect, they also reduce costs effectively.

However, as we mentioned previously, different hydrogel‐formed materials, different demands, and different applications usually lead to specific limitations that need to be solved. Herein, we anticipated the development trends in the future.

1. Multifunctionalization. Smart hydrogels that incorporate multiple functions have been increasingly developed. In addition to delivering therapeutic agents such as stem cells and anti‐inflammatory drugs, these hydrogels could also be developed to simultaneously achieve MRI, CT, fluorescence imaging, repair or serve as substitute for damaged tissues, create a suitable environment for the growth and differentiation of stem cells, dual drug delivery, immune regulation, antibacterial, combination therapy, etc. There also researchers combined these smart hydrogels with wearable devices or smart phones to realize real‐time monitor. Investigation of multiresponsive hydrogel is also an ideal strategy to achieve multifunctionalization.
2. Development of new stimuli factors. The development of new stimulating factors can effectively improve the targeting capacity and response sensitivity of smart hydrogels. For endogenous stimuli factors, researchers have investigated a great deal of novel stimuli factors for specific inflammation management. Among them, the most studied types are enzymes that are present in the IMEs or produced by pathogens. In Subsection 4.4, we summarized a number of examples of treating inflammation in response to specific enzymes, all of which have enabled more precise local treatment. For external factors, there are also many emerging types according to different requirements, such as electric‐responsive and shear force‐responsive smart hydrogels, both of them are aiming at better controllability and convenience.
3. Development of novel biomaterials and improvement of existing biomaterials. In addition to the modification and cross‐linking of existing materials, the development of new materials is also indispensable. In the past decade, a large number of biomaterial derivative‐based hydrogels, block or graft copolymer‐based hydrogels and cross‐linked hydrogels have been designed for a wide variety of applications. As for emerging biomaterials, represented by nucleic acid and peptide, more and more new biomaterials have been developed for hydrogel preparation to treat diseases. Whether developing new materials or improving existing materials, there are three basic conditions that should be met: i) favorable biosafety, ii) effectiveness, and iii) lower cost and easy to produce.
4. Promotions for clinical translation. In the process of clinical translation, in addition to the possible side effects which could be caused by smart hydrogels, many essential factors should be considered simultaneously, such as patient compliance, convenience, and economy. To date, clinical test of several kinds of biomaterial‐based hydrogel are ongoing, such as GLY‐200, VentriGel, and IK‐5001. Besides, there some biomaterials like PEG, PAA, HA, and SA, though no hydrogel production are testing, there biosafety has been verified in medical, food, and cosmetic processing, hydrogel made by these materials also have a promising future to realize clinical translation in the next decades. Nevertheless, although plenty of studies have achieved satisfactory results, its real application in the treatment of inflammations still needs more rigorous experimental evaluation, and needs to be designed in conjunction with their case scenarios of purpose uses. For example, stimulus‐responsive hydrogels could be designed as pepper spray for the treatment of inflammation caused by upper respiratory tract infection, and which could also be designed as eye drops to treat ocular inflammation for improving patient compliance of drug carriers through dosage form design in their clinical transformation researches.
5. Further pathology research. The development of stimulus‐responsive hydrogels for the treatment of various inflammations is a good example of using engineering principles to solve medical problems. Furthermore, the systematic summary of the above‐mentioned kinds of hydrogels was made to clearly show their merits, properties, and applications (**Table** **8**). As an emerging interdisciplinary field, its development must be inseparable from the progress of medicine and pharmacology. Nowadays, the pathogenesis of many inflammations is still unclear and most of the clinical treatment for them is primarily focused on symptom alleviation. Therefore, in order to achieve effective treatment of these diseases, more researchers are expected to dedicate themselves to the study and exploration of the pathogenesis. With the development of medicine, materials science, electronics, and so on, smart hydrogels could be the next generation platforms for inflammatory treatment.

**Table 8:** Merits, properties, and applications of the hydrogels for inflammation treatment.

Types	Agents	Examples	Refs.	Features and physicochemical characteristics
Natural biomaterials	Chitosan	Carboxymethyl chitosan (CMCS) Thiolated chitosan (TCS) Chitosan–alginate (CA)	[75] [78] [185]	The only natural cationic polysaccharide. Economical because of its large existence in nature. Good mechanism properties and less toxicity. Certain ability to suppress bacteria growth. Suitable for pH‐responsive hydrogel preparation due to the solubility difference at different pH. Easier to be modified and functionalized.
	Hyaluronic acid	Methacrylated hyaluronic acid (HAMA) EGF@PR‐S gel	[220] [249]	Natural polysaccharide with good biocompatibility. Crucial ingredient to make up the human body. High productivity. More matured products have been applied.
	Sodium alginate	pH‐sensitive SA‐cellulose‐based hydrogel	[95]	Favorable biocompatibility, mucoadhesiveness, degradability. Inherent pH‐sensitivity.
	Dextran	Dextran‐based pH‐sensitive nanogel	[106]	Helpful in blood glucose regulation and cholesterol metabolism. Enhance immunity. Capacity to suppress bacteria and tumor growth. Good biocompatibility. Easy to be modified or cross‐linked.
	Cellulose	Bacteria cellulose	[190]	The most abundant polysaccharide on earth. Easy to be cross‐linked or react with modification agents. With good stability and mechanical property because of intermolecular hydrogen bond. Good biodegradability and renewability.
	Gelatin	GelMA	[124] [126]	Strong hydrophilicity and low cost. Could be modified with various ions or groups. Good biocompatibility.
	Guar gum	GG‐based pH‐sensitive hydrogel	[132]	Extraction of Cyamopsis tetragonoloba. Low toxicity, green, economical. Easy to be modified and cross‐linked. Often‐used in food industry.
	Peptide	Jelleine‐1 KEF9 FBA‐VKVKVK	[133] [134] [210]	Widely exists in organisms and important in physiological activity. Inherent biocompatibility, biodegradability, bioactivity, and biosafety. Form hydrogel through self‐assemble. Easier to be functionalized and modified.
Synthetic biomaterials	PLA	N_3_–PEG–PLA	[204]	Nontoxicity, biodegradability, and environmentally friendly. Have been applied in various fields. Could not form hydrogel alone. Endow hydrogel with thermosensitivity.
	PAAm	pNiPAAm	[144]	High water‐holding capacity and good thermostability. Controllable physiochemical properties. Widely used in copolymer preparation.
	PAA	Alginate‐graft‐PAA pH‐sensitive hydrogel	[156]	High water‐holding capacity. Favorable optical capacity and solubility. Good biodegradability. Easy to be modified due to abundant ─COOH. pH sensitivity.
	PEG	PLGA–PEG–PLGA	[162]	Widely used in various fields and productions. Excellent biocompatibility. Often‐used for surface modification and block copolymer design.
	PVA	PVA–gelatin hybrid hydrogel	[147]	Widely used in various fields and productions. PVA‐modified hydrogels possess preferred toughness and strength. Usually worked as a modification agent or cross‐linker.
	PNP	PNP‐based injectable thermosensitive hydrogel	[148]	Excellent biodegradability and biocompatibility. Insolubility. Controllable glass‐transition temperature.
	Block copolymer	P407 DMA–MPC	[174] [127]	Excellent design flexibility. Possess different properties due to different constituent.

## Conflict of Interest

The authors declare no conflict of interest.

## Data Availability

All relevant data are within the paper. The data are available from the corresponding author on reasonable request.
